# Cardiac-specific microRNA-125b deficiency induces perinatal death and cardiac hypertrophy

**DOI:** 10.1038/s41598-021-81700-y

**Published:** 2021-01-27

**Authors:** Chen-Yun Chen, Desy S. Lee, Oi Kuan Choong, Sheng-Kai Chang, Tien Hsu, Martin W. Nicholson, Li-Wei Liu, Po-Ju Lin, Shu-Chian Ruan, Shu-Wha Lin, Chung-Yi Hu, Patrick C. H. Hsieh

**Affiliations:** 1 Cardiovascular Division, Institute of Biomedical Science, Academia Sinica, National Taiwan University College of Medicine, 128 Academia Road, Sec. 2, Nankang, Taipei, 115 Taiwan; 2 Department of Clinical Laboratory Sciences and Medical Biotechnology, College of Medicine, National Taiwan University, Taipei, 100 Taiwan; 3 Department of Biomedical Sciences and Engineering, National Central University, Taoyuan, 320 Taiwan; 4 Institute of Medical Genomics and Proteomics, National Taiwan University College of Medicine, Taipei, 100 Taiwan; 5 Institute of Clinical Medicine, National Taiwan University College of Medicine, Taipei, 100 Taiwan

**Keywords:** Developmental biology, Molecular biology, Physiology

## Abstract

MicroRNA-125b, the first microRNA to be identified, is known to promote cardiomyocyte maturation from embryonic stem cells; however, its physiological role remains unclear. To investigate the role of miR-125b in cardiovascular biology, cardiac-specific miR-125b-1 knockout mice were generated. We found that cardiac-specific miR-125b-1 knockout mice displayed half the miR-125b expression of control mice resulting in a 60% perinatal death rate. However, the surviving mice developed hearts with cardiac hypertrophy. The cardiomyocytes in both neonatal and adult mice displayed abnormal mitochondrial morphology. In the deficient neonatal hearts, there was an increase in mitochondrial DNA, but total ATP production was reduced. In addition, both the respiratory complex proteins in mitochondria and mitochondrial transcription machinery were impaired. Mechanistically, using transcriptome and proteome analysis, we found that many proteins involved in fatty acid metabolism were significantly downregulated in miR-125b knockout mice which resulted in reduced fatty acid metabolism. Importantly, many of these proteins are expressed in the mitochondria. We conclude that miR-125b deficiency causes a high mortality rate in neonates and cardiac hypertrophy in adult mice. The dysregulation of fatty acid metabolism may be responsible for the cardiac defect in the miR-125b deficient mice.

## Introduction

Cardiac hypertrophy is a serious health risk which can lead to heart failure. Cardiac hypertrophy is an adaptive response to hemodynamic stress^1^ resulting from, for example, excess exercise, pregnancy or due to valvular disease, and may ultimately lead to cardiomyopathy and heart failure^2–4^. Previous studies have indicated that microRNAs (miRNA), a class of small non-coding RNA, is important for transcriptional and post-transcriptional inhibition of gene expression^5^, play an important role in the development of cardiomyopathy. Cardiomyocyte-specific deletion of Dicer (*dgcr8*), an enzyme required for the processing of miRNA^6^, revealed a fully penetrant phenotype that begins with left ventricular malfunction progressing to a dilated cardiomyopathy and premature lethality^7^. In another study, cardiac-specific Dicer knockout mice showed perinatal death with dilated cardiomyopathy^8^. In most cases, miRNA knockouts are viable, fertile, and seemingly normal with only modest phenotypes^9^; however, these studies show that cardiac-specific knockout of miRNA-processing enzymes leads to cardiomyopathy.

MicroRNA-125b (miR-125b), an orthologue of lin-4 in *C. elegans*, was the first miRNA to be discovered^10^. Since then, miR-125b has been shown to be an important regulator of developmental timing in *C. elegans*^10^. Previous work in our lab showed that miR-125b, combined with other three miRNAs, promotes the maturation of both mouse and human embryonic stem cell-derived cardiomyocytes (CM)^11^. Another study employing CM showed that the β-blocker carvedilol promotes cardioprotection through miR-125b by inhibition of the pro-apoptotic genes Bak1 and Klf13^12^. Additionally, miR-125b has been shown to protect the heart from ischemic injury^13^. Increased expression of miR-125b attenuates both hypoxia/reoxygenation (H/R) and ischaemia/reperfusion (I/R)-induced cell injury and cell death in H9C2 cardiomyoblasts and adult cardiac myocytes^13^. In contrast, miR-125b acts as a potent repressor of multiple anti-fibrotic mechanisms thus plays a role in the induction of cardiac fibrosis^14^. However, despite previous efforts, the biological role of miR-125b in maintaining heart function remains unknown.

In the present study, we examined the role of miR-125b in the heart and show that knockout of cardiac-specific miR-125b induces cardiac hypertrophy, cardiac impairment due to disrupted mitochondrial dynamics and function and prenatal lethality. Therefore, we demonstrate that miR-125b plays a vital and multifaceted role in maintaining healthy heart function and development.

## Results

### Cardiac-specific knockout of miR-125b-1 induces perinatal lethality

**Figure 1:**
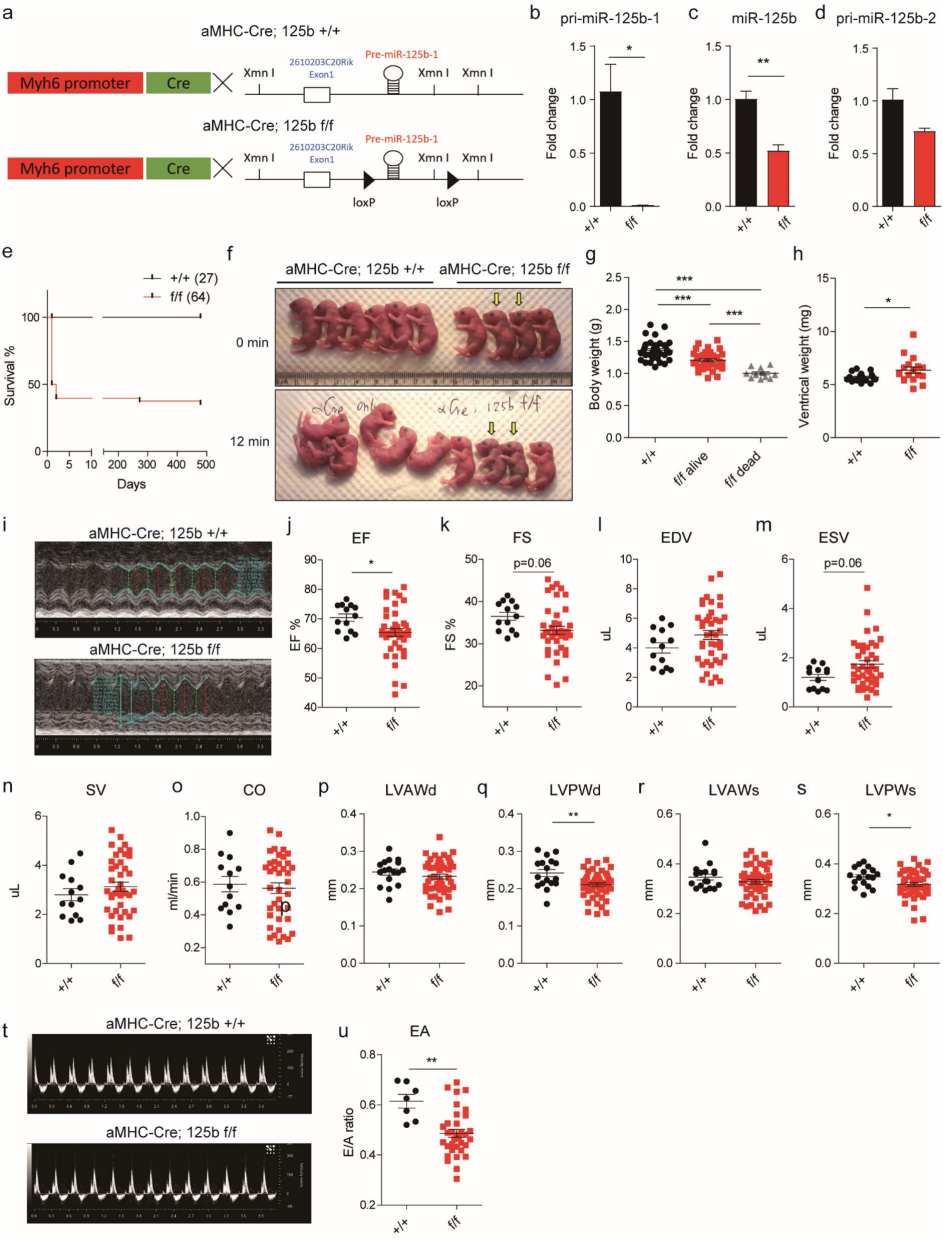
Cardiac-specific knockout of miR-125b-1 increased perinatal lethality and impaired systolic and diastolic heart function. (**a**) The design of the Cre-loxp system. The miR-125b-1 chromosome locus was flanked by loxp sequence. In the following definition, + / + indicates Myh6-cre; miR125b1 + / + and f/f indicates Myh6-cre; miR125b1f./f. (**b**) The expression of primary miR-125b-1 transcript by qPCR in the control and knockout neonatal ventricles (n = 3 in each group, *p* = 0.0142). (**c**) The expression of mature miR-125b by qPCR in the knockout neonatal ventricles (n = 3 in each group, *p* = 0.0062). (**d**) The expression of primary miR-125b-2 transcript by qPCR in the control and knockout neonatal ventricles (n = 3 in each group, *p* = 0.005). (**e**) The survival curve of cardiac-specific miR-125b-1 knockout mice (n = 27 vs. 64 in control and knockout mice). (**f**) The appearance of knockout neonates. (**g**) The body weight of control and knockout neonates (n = 34, 37 and 11 in the + / + control, f/f alive knockout and f/f dead knockout mice, one-way ANOVA with Tukey’s post-test was used. Adjust *p* < 0.001 in all comparisons). (**h**) The ventricular weight of control and knockout neonates (n = 18 vs. 17, *p* = 0.0388). (**i**) Echocardiography of neonatal hearts with M-mode view. (**j**) Ejection fraction in the neonatal left ventricles (n = 13 vs. 41, *p* = 0.0428). (**k**) Fraction shortening in the neonatal left ventricles (n = 13 vs. 41, *p* = 0.0633). (**l**) End-diastolic volume in the neonatal left ventricles (n = 13 vs. 41, *p* = 0.1353). (**m**) End-systolic volume in the neonatal left ventricles (n = 13 vs. 41, *p* = 0.0557). (**n**) Stroke volume (n = 13 vs. 41, *p* = 0.3642) and (**o**) cardiac output in the neonatal left ventricles (n = 13 vs. 41, *p* = 0.6975). (**p**) Left ventricular end-diastolic anterior wall thickness (n = 17 vs. 47, *p* = 0.3223). (**q**) Left ventricular end-diastolic posterior wall thickness (n = 17 vs. 47, *p* = 0.0038). (**r**) Left ventricular end-systolic anterior wall thickness (n = 17 vs. 47, *p* = 0.2640). (**s**) Left ventricular end-systolic posterior wall thickness (n = 17 vs. 47, *p* = 0.0415). (**t**) M-mode of mitral inflow by Doppler image in the neonatal left ventricles. (**u**) The velocity ratio of early filling (E wave) and late diastolic filling (A wave) in the neonatal hearts (n = 7 vs. 34, *p* = 0.0014). The significance of the differences between control and knockout groups were determined by unpaired Student *t*-test.

To define miR-125b function in mammalian hearts, we targeted the genome sequence of miR-125b-1 by homologous recombination in mouse embryonic stem (ES) cells. The miR-125b-1 resides in the transcript of an unknown gene 2610203C20Rik and the distance between the two loxP sites is about 1.49 kb (Fig. 1a). Given that mir-125b-1 resides in intron1 of 2610203C20Rik, a Mir100-Mirlet7a-2-Mir125b-1 cluster host gene, we examined the 2610203C20Rik expression level by qPCR in mir-125b1 f./ + mice and their mir-125b1 + / + littermates control. There was no difference between mir-125b1 f./ + and -125b1 + / + , indicating that the flox strategy would not affect the transcription of 2610203C20Rik locus and its intronic miR-125b-1 (Fig. S1a). In addition, no differential expression was found in the neighboring genes *let-7a* and *Ubash3b* either (Fig. S1b and c). Regarding the phenotypes, littermates with different genotypes displayed normal heart weight- to-body weight ratio in adults, except cardiac specific miR-125b-1 knockout mice (Fig. S1d). Thus, we conclude that there was no evidence showing the flox strategy we used would affect 2610203C20Rik or miR-125b-1 expression or the cardiac phenotypes substantially. The knockout efficacy of pri-miR-125b-1, by cardiac-specific Cre driven by Myh6 promoter was 99% in the neonatal hearts (Fig. 1b), which resulted in a reduction of 50% mature miR-125b (Fig. 1c). The remaining mature miR-125b could be derived from miR-125b-2, since the pri-miR-125b-2 was expressed in the hearts (Fig. 1d). It was noticed that miR-125b was not only detected in cardiomyocytes but also in the endothelial cells, leukocytes and fibroblasts (Fig. S2). The remaining mature miR-125b could also be from other cell types. The knockout efficacy of pri-miR-125b-1 in neonatal and adult hearts was highly efficient even without cardiomyocyte isolation, which indicated that the expression of pri-miR-125b-1 was predominant in cardiomyocytes (Figs. 1b, 4d). Half of the knockout mice died within two days and a few mice suddenly died in adulthood (Fig. 1e). The knockout neonates were weaker than the wild-type mice. For example, if the neonates were aligned on tissue paper, wild-type mice moved around 12 min later but the knockout mice barely moved, some of them just died (Fig. 1f). The knockout neonates were lighter than the wild-type mice (Fig. 1g). Further, we measured the ventricular weight and found that the ventricular weight of knockout neonates was higher than that of the wild-type mice (Fig. 1h). Cardiac-specific knockout of miR-125b-1 led to perinatal death and increased ventricular weight.

To examine the heart function of neonatal hearts, we used high-frequency echocardiography with Doppler (Fig. 1i). It showed that the ejection fraction and fraction shortening decreased in the knockout neonates (Fig. 1i, j, k) without significant change in end diastolic volume (EDV), end systolic volume (ESV), stroke volume (SV) and cardiac output (CO) (Fig. 1l–o). The posterior wall thickness on echocardiography was reduced at end diastole (LVPWd) and end systole (LVPWs) (Fig. 1q, s), and the anterior wall thickness (LVAWd and LVAWs) was normal (Fig. 1p, r). Interestingly, we observed a reduction of E/A ratio in the knockout mice (Fig. 1t, u), which indicated diastolic dysfunction. MiR-125b deficiency-induced cardiac hypertrophy and impaired systolic and diastolic function in neonatal hearts.

### MiR-125b deficiency alters mitochondria morphology, dynamics and function

**Figure 2:**
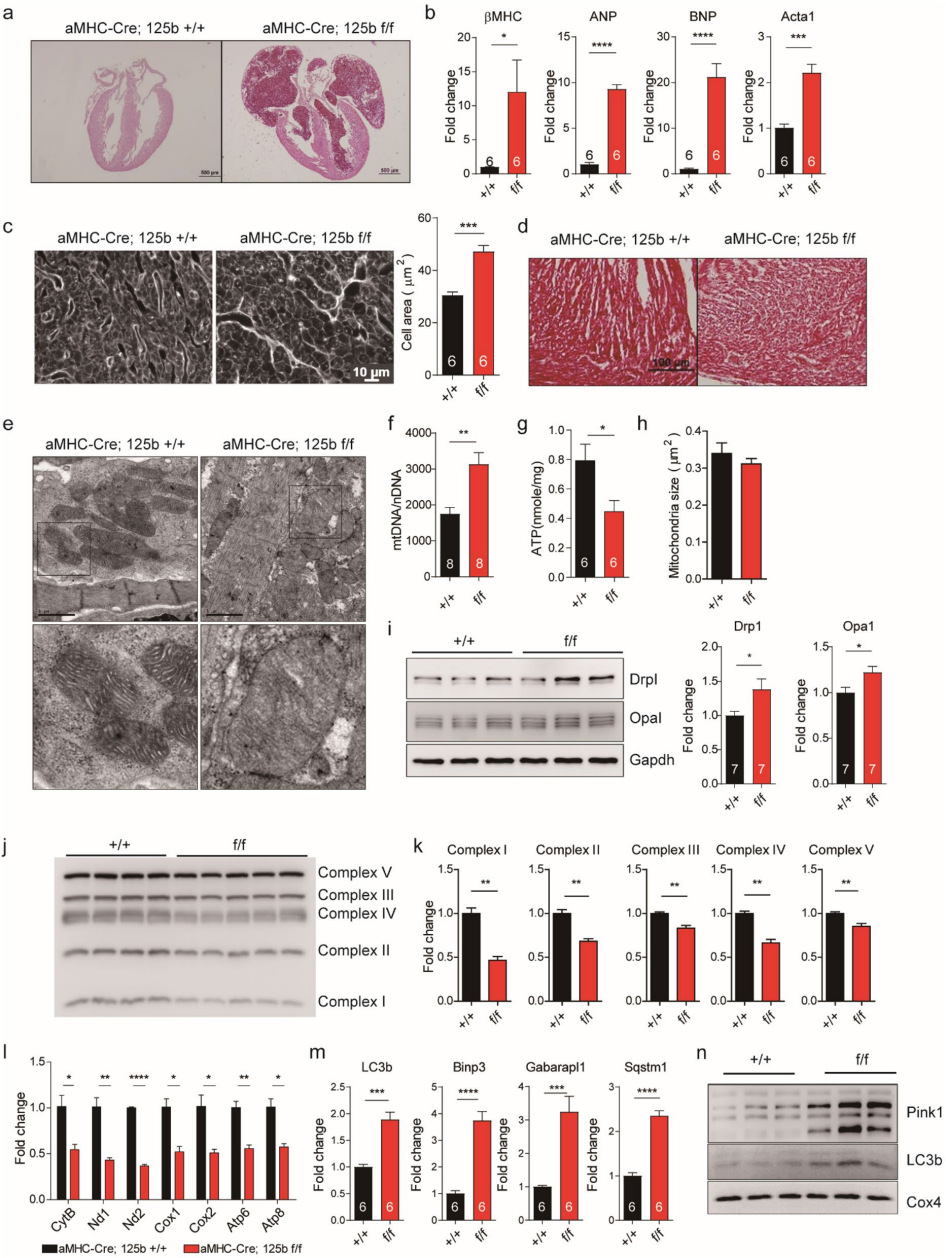
MiR-125b deficiency induces cardiac hypertrophy and alters mitochondria morphology, dynamics and function. (**a**) HE staining of neonatal (P1) hearts. (**b**) βMHC, ANP, BNP and Acta1 gene expression in the neonatal ventricles (n = 6 for each group, βMHC *p* = 0.0418, ANP *p* < 0.0001, BNP *p* < 0.0001, Acta1 *p* = 0.0002). (**c**) Wheat germ agglutinin (WGA) staining of the neonatal myocardium and the quantification of cardiomyocyte size (n = 6 for each group, *p* = 0.0001). (**d**) Trichrome stain of the neonatal myocardium. Bar: 100 µm. (**e**) TEM of the neonatal myocardium. Bar: 1 µm. (**f**) The ratio of mitochondrial DNA copy number to nuclear genome DNA copy number (n = 8 for each group, *p* = 0.0025). (**g**) ATP amount in neonatal hearts (n = 6 for each group, *p* = 0.0280). (**h**) Mitochondrial size (n = 84 vs. 166 from 3 mice for each group, *p* = 0.3400). (**i**) Drp1 and Opa1 expression and blot quantification. Drp1 and Gapdh were cropped from the same blot. Opa1 was from the other blot with the same protein extracts in Drp1 blot. To increase the n number, another experimental repeat was conducted (Fig. S8). Protein quantification was based on these two experiments (n = 7 for each group, Drp1 *p* = 0.0343, Opa1 *p* = 0.0302). (**j**) The expression of OXPHOS complexes in the isolated mitochondria. (**k**) The quantification of OXPHOS complexes (n = 4 vs. 5, Complex I *p* = 0.0028, Complex II *p* = 0.0011, Complex III *p* = 0.0022, Complex IV *p* = 0.0053, Complex V *p* = 0.0064). (**l**) Mitochondrial encoded gene expression in the knockout myocardium (n = 3 for each group, CytB *p* = 0.0253, Nd1 *p* = 0.0049, Nd2 *p* < 0.0001, Cox1 *p* = 0.0109, Cox2 *p* = 0.0178, Atp6 *p* = 0.0045, Atp8 *p* = 0.0117). (**m**) The gene expression of LC3b, Binp3, Gabarapl1 and Sqstm1 (n = 6 for each group, LC3b *p* = 0.0001, Binp3 *p* < 0.0001, Gabatapl1 *p* = 0.0007, Sqstm1 *p* < 0.0001). (**n**) The expression of Pink1 and LC3b in the isolated mitochondria. Pink1, LC3b and Cox4 were from different blots with the same protein extracts. The original images of i, j and n are displayed in Fig. S8. The significance of the differences between control and knockout groups were determined by unpaired Student *t*-test.

The histology of the knockout mice showed enlarged hearts in dead neonates, especially the atrium (Fig. 2a). Atrial enlargement is common in heart failure with preserved ejection fraction (HFpEF) patients and is a risk factor for advanced heart failure^15^. In addition, the stress markers including βMHC, ANP, BNP, and Acta1 were upregulated in the knockout neonatal hearts (Fig. 2b). Cardiomyocyte size was then quantified. The result showed the size of cardiomyocytes in the knockout mice was significantly larger than that in the wild type (Fig. 2c). We did not observe fibrosis in the neonatal hearts by Masson trichrome staining; however, we noticed a loose myocardium structure (Fig. 2d).

Under transmission electron microscopy (TEM), myofibrils in the knockout cardiomyocytes had become less compact and the mitochondria had less cristae (Fig. 2e). Further, we investigated the changes in mitochondria by measuring the amount of mitochondria, ATP production, and mitochondria size. Although the copy number of mitochondria increased (Fig. 2f), the ATP production was reduced by half (Fig. 2g) without changing the size of the mitochondria (Fig. 2h). In addition, both fission protein Drp1 and fusion protein Opa1 were upregulated in the knockout mice (Figs. 2i, S8, S9), which indicated miR-125b deficiency accelerated the dynamics of mitochondria fission and fusion. There were much fewer mitochondria complex proteins (Figs. 2j, k, S8) and mitochondria encoded gene transcripts (Fig. 2l) in the isolated mitochondria from the knockout mice. MiR-125b deficiency impaired the transcription and protein translation machinery. Mitophagy related genes LC3b, Binp1, Gabarapl1, and Sqstm1 were upregulated in the knockout mice (Fig. 2m). Western blot of isolated mitochondria proteins also showed more Pink1 and LC3b (Figs. 2n, S8), which suggested activation of mitophagy in miR-125b deficient mice.

### Adult miR-125b deficient mice displayed impairment of heart function

**Figure 3:**
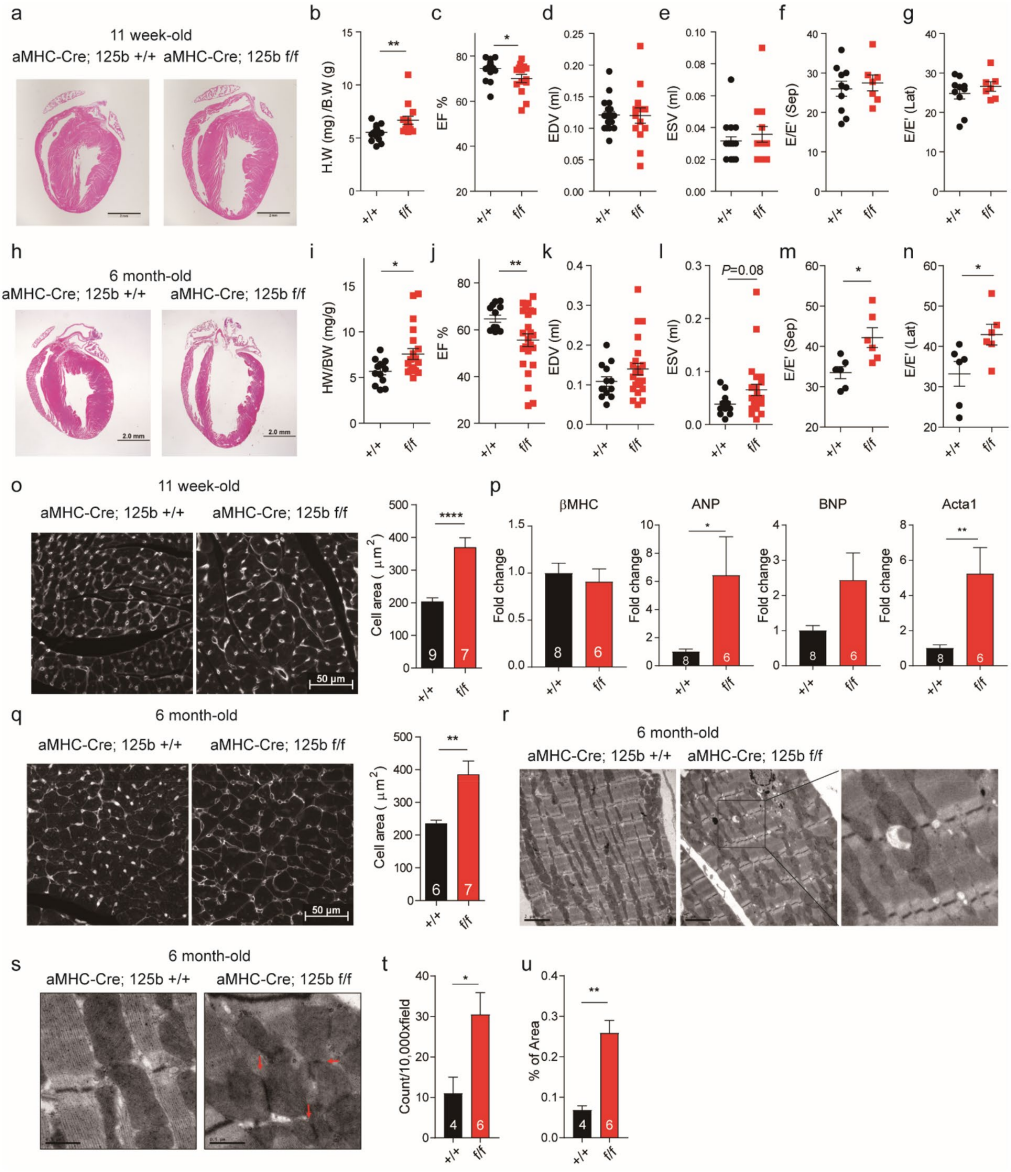
MiR-125b deficiency promotes the progression of heart failure. (**a**) HE staining of young adult hearts. Bar: 2 mm. (**b**) Heart weight-to-body weight ratio of young adult mice (n = 19 vs. 14, *p* = 0.0044). (**c**) Ejection fraction (n = 19 vs.. 14, *p* = 0.0262), (**d**) end-diastolic volume (n = 19 vs.. 14, *p* = 0.1211) and (**e**) end-systolic volume (n = 19 vs.. 14, *p* = 0.4270) in young adult mice. (**f**) The ratio of early diastolic mitral inflow velocity to early diastolic septal mitral annulus velocity (E/E' ratio) (n = 19 vs. 14, *p* = 0.06049) and (**g**) the ratio of early diastolic mitral inflow velocity to early diastolic lateral mitral annulus velocity (E/E' ratio) (n = 19 vs. 14, *p* = 0.3833) in young adult mice. (**h**) HE staining of mature adult mice. Bar: 2 mm. (**i**) Heart weight-to-body weight ratio of mature adult mice (n = 10 vs. 18, *p* = 0.0358). (**j**) Ejection fraction (n = 10 vs. 18, *p* = 0.0077), (**k**) end-diastolic volume (n = 10 vs. 18, *p* = 0.1489) and (**l**) end-systolic volume (n = 10 vs. 18, *p* = 0.0894) in mature adult mice. (**m**) The ratio of early diastolic mitral inflow velocity to early diastolic septal mitral annulus velocity (E/E' ratio) (n = 6 vs. 6, *p* = 0.0132) and (**n**) the ratio of early diastolic mitral inflow velocity to early diastolic lateral mitral annulus velocity (E/E' ratio) (n = 6 vs. 6, *p* = 0.0353) in mature adult mice. (**o**) Quantification of cardiomyocyte size by WGA staining in the 11-week-old adult myocardium (n = 9 vs. 7, *p* < 0.0001). (**p**) βMHC, ANP, BNP and Acta1 gene expression in the young adult myocardium (n = 8 vs. 6, βMHC *p* = 0.5999; ANP *p* = 0.0395, BNP *p* = 0.0587, Acta1 *p* = 0.0069). (**q**) Quantification of cardiomyocyte size by WGA staining in the 6-month-old adult myocardium (n = 6 vs. 7, *p* = 0.0063). (**r**) TEM of the 6-month-old adult myocardium. Bar: 2 µm. (**s**) TEM of the 6-month-old adult myocardium with electron-dense mitochondrial contact sites, indicated by red arrows. Bar: 0.1 µm. (**t**) Numbers of droplets under 10,000 × field by TEM (n = 4 vs. 6, *p* = 0.0306). (**u**) Percentage of droplet area in tissues under 10,000 × field by TEM (n = 4 vs. 6, *p* = 0.0015). The significance of the differences between control and knockout groups were determined by unpaired Student *t*-test.

Mice that survived to young adulthood (2–3 months old) behaved normally but myocardium enlargement was observed (Fig. 3a, b). Measurement of cardiomyocyte size also demonstrated that the cell size in the knockout mice was larger than that in the wild type (Fig. 3o). The stress markers ANP and Acta1 were upregulated in the knockout young adult hearts (Fig. 3p). Echocardiography showed a reduced ejection fraction in the knockout mice with modest difference (Fig. 3c). No significant differences were found in the EDV, ESV and E/E′ ratio between control and knockout mice (Fig. 3d–g). Therefore, β-adrenergic agonist isoproterenol was used to induce cardiac hypertrophy. The result showed that under the treatment, the heart weight-to-body weight ratio of knockout mice was significantly larger than that of the wild-type mice (Fig. S3a), and the ejection fraction was reduced significantly (Fig. S3b). Both EDV and ESV showed a more pronounced increasing trend in the knockout mice (Fig. S3c, d).

In mature adult mice (6 months old), the knockout mice presented dilated cardiomyopathy (DCM) (Fig. 3h) and significantly increased heart weight-to-body weight ratio (Fig. 3i) and cardiomyocyte size (Fig. 3q). The ejection fraction was reduced and the E/E′ ratio increased significantly (Fig. 3j, m, n). Both EDV and ESV showed an increasing trend in the knockout mice (Fig. 3k, l). The E/E′ ratio is a recommend method for the evaluation of diastolic function in the clinic^16^. Elevated E/e′ ratio is considered to be a prognostic factor for the development of cardiac vascular disease^17^. The increase in E/E′ ratio indicated a diastolic dysfunction in the mature knockout hearts. We further evaluated the heart function by cardiac catheterization. Interestingly, we found that the heart rates and the end systolic pressure were significantly higher than the wild type (Fig. 6e, f). Unexpectedly, both max dP/dt and min dP/dt were better than the wild-type mice (Fig. 6g, h), which may be influenced by heart rates and end systolic pressure^18^. For example, doxorubicin suppressed the left ventricular systolic pressure and influenced the systolic (max dP/dt) and diastolic (min dP/dt) indicators^19^. Physiologically, heart rate is regulated by epinephrine from the adrenal medulla and norepinephrine from the sympathetic nerve terminals^20^. MiR-125b can be detected in exosomes^21,22^, and possibly be delivered to kidney or brain from the heart. Notwithstanding, the regulatory role of miR-125b in the production of epinephrine or norepinephrine is still unknown. Blood pressure regulation depends on the renin–angiotensin–aldosterone system (RAAS). RAAS-related gene ACE, angiotensin I converting enzyme, is the potential target of miR-125b according to TargetScan^23^ prediction and miR-125b was expressed in arteriolar vascular smooth muscle cells and in juxtaglomerular cells, renin producing cells, in kidney^24^. MiR-125b in kidney may have a negative regulatory role in blood pressure regulation and such a role could be Tp53 dependent. Another possibility is that miR-125b has an impact on baroreceptor, a blood pressure sensor, in hearts^25^ and causes the heart rate and blood pressure dysregulation. No difference was found in relaxation time constant (Tau), end-systolic PV relation slope (ESPVR slope) and end-diastolic PV relation slope (EDPVR slope) (Fig. 6i–k) between the control and the knockout mice, which suggests the deficiency in cardiac contractility and compliance in the knockout mice was obscure. In the mature knockout mice, we found misaligned sarcomeres and mitochondria (Fig. 3r). In addition, several vesicles, possibly lipid droplets, were also obvious (Fig. 3r). The number of lipid droplets and the area of lipid droplets were quantified by ImageJ (Fig. 3t, u). Both showed significant increases in in the knockout mice. Some fission-like mitochondria were also found in the knockout mice (Fig. 3s). The mature knockout mice not only showed DCM with increased cardiomyocytes but also showed abnormal intracellular organization.

### Mitochondrial miR-125b were dysregulated in cardiac miR-125b deficient mice

**Figure 4:**
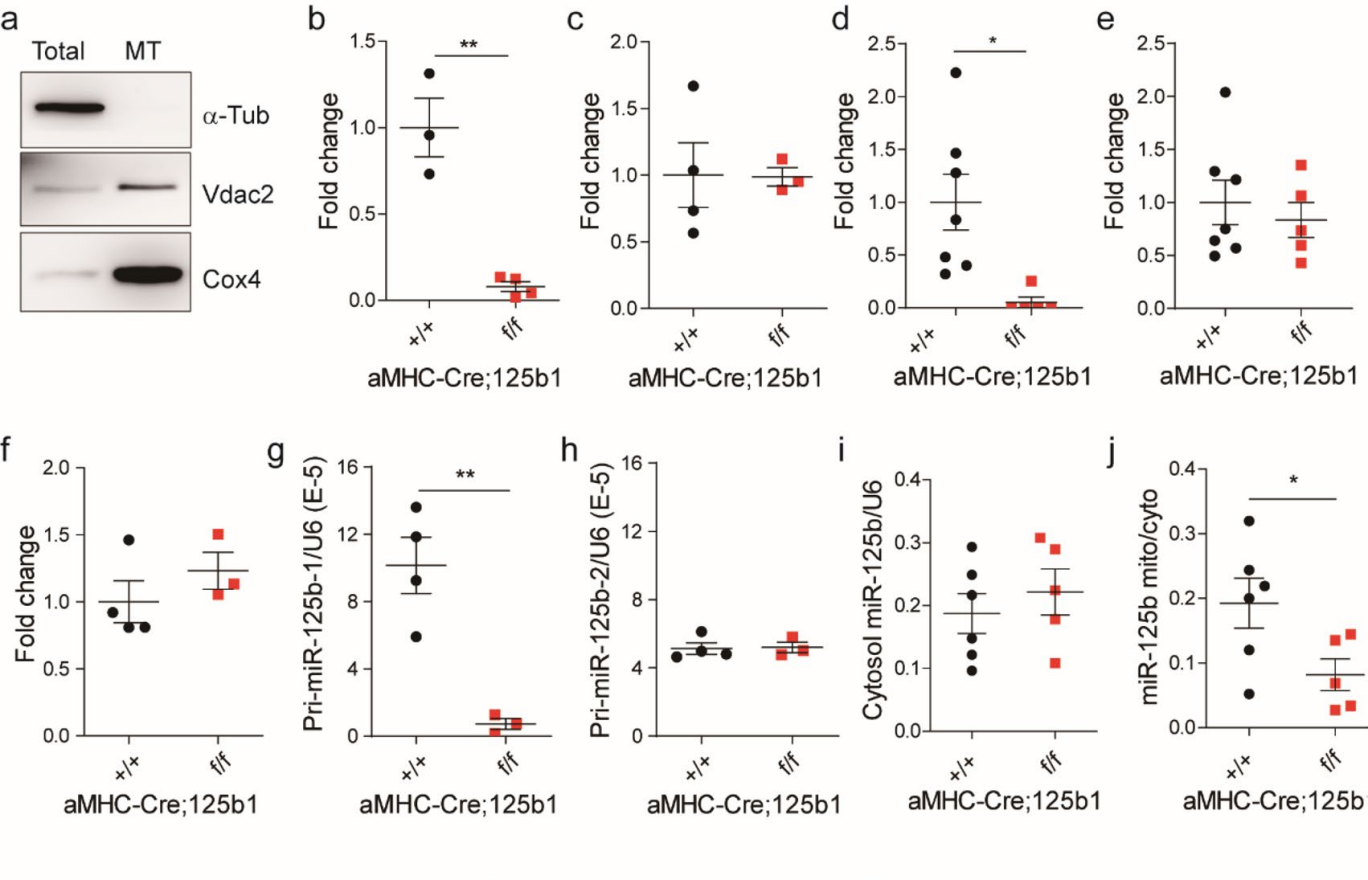
Mitochondrial miRNA-125b expression in neonatal and adult hearts. (**a**) Confirmation of isolated mitochondria from neonatal hearts. Vdac2 and Cox4 are mitochondrial proteins and αtubulin is a cytosolic protein. MT: mitochondria. Vdac2, Cox2 and αtubulin were cropped from the same blot. The original images of blot were showed in Fig. S7. (**b**) Mitochondrial miRNA-125b expression in neonatal hearts. A spike-in control, cel-mir-39, was used to normalize the technical error (n = 3 vs. 4, *p* = 0.0015). (**c**) MiRNA-125b expression in young adult myocardium (n = 4 vs. 3, *p* = 0.9667). (**d**) Pri-miR-125b-1expression in young adult myocardium (n = 7 vs. 5, *p* = 0.0139). (**e**) Pri-miR-125b-1expression in young adult myocardium (n = 7 vs. 5, *p* = 0.5758). (**f**) Pri-miR-125b-1 expression in isolated cardiomyocytes from young adult myocardium (n = 4 vs. 3, *p* = 0.0053). (**h**) Pri-miR-125b-2 expression in isolated cardiomyocytes from young adult myocardium (n = 4 vs. 3, *p* = 0.8961). (**i**) Cytosolic miR-125b expression in young adult hearts (n = 6 vs. 5, *p* = 0.1874). (**j**) The ratio of mitochondrial miR-125b to cytosolic miR-125b in young adult hearts (n = 6 vs. 5, *p* = 0.0470). The significance of the differences between control and knockout groups were determined by unpaired Student *t*-test.

The knockout mice that survive to adulthood might not exhibit the pathological phenotype in the young adult period but gradually develop cardiac hypertrophy later. However, we noticed that the total mature miR-125b in the knockout myocardium returned to the normal level (Fig. 4c). We also isolated cardiomyocytes from the young adult hearts and found the expression level of mature miR-125b in the knockout cardiomyocytes was similar to that in the wild type (Fig. 4f). The knockout efficiency of pri-miR-125b-1 was still superior in adult myocardium and in isolated cardiomyocytes (Fig. 4d, g). Therefore, the origin of miR-125b in the knockout mice could be pri-miR-125b-2, although it did not show significant upregulation in adult myocardium and in isolated cardiomyocytes for the rescue (Fig. 4e, h). MiR-125a belongs to the miR-125 family, but it was undetectable in hearts. The expression change of pri-mir-125b-1 and pri-mir-125b-2 in isolated cardiomyocytes (Fig. 4g, h) was similar to that in the myocardium (Fig. 4d, e) but the basal level of pri-mir-125b-1 was two-fold higher than pri-mir-125b-2, which implies cardiomyocytes prefer to use pri-mir-125b-1 as the source of miR-125b. If the function of miR-125b could be compensated by pri-mir-125b-2, it raises an issue about the relationship between the adult phenotype and the expression of miR-125b. One possibility is that adult cardiomyopathy is determined at the neonatal stage, and the other possibility is that the miRNA has unexplored functions. Since the knockout of miR-125b changes energy homeostasis, we investigated its expression in mitochondria. Surprisingly, the downregulation of mitochondrial miR-125b (mito-miR-125b) was more obvious than the downregulation of mature miR-125b in the knockout neonatal hearts (Figs. 1c and 4b). In adults, the mito-miR-125b was also decreased in the knockout myocardium (Fig. 4j), although the cytosolic miR-125b was unchanged (Fig. 4i). The function of mitochondrial miRNAs (mitomiRs) is still unclear. A summary of microRNAs found in the mitochondria has been presented previously^26,27^, In particular miR-125b has been identified in many studies^28,29^. In diabetic hearts, mito-miR-125b was downregulated^29^, which emphasizes the strong link between miR-125b and cardiac metabolism. There are several possible mechanisms. For example, mito-miR-1 promotes mitochondrial translation in differentiated myofibroblats^30^. A recent study also suggests that mito-miR-2392 inhibits mitoDNA transcription instead of translation^31^. The function of mito-miR-125b and how miR-125b was transported into the mitochondria require further investigation.

### Transcriptome and proteome analysis of cardiac miR-125b deficient mice

**Figure 5:**
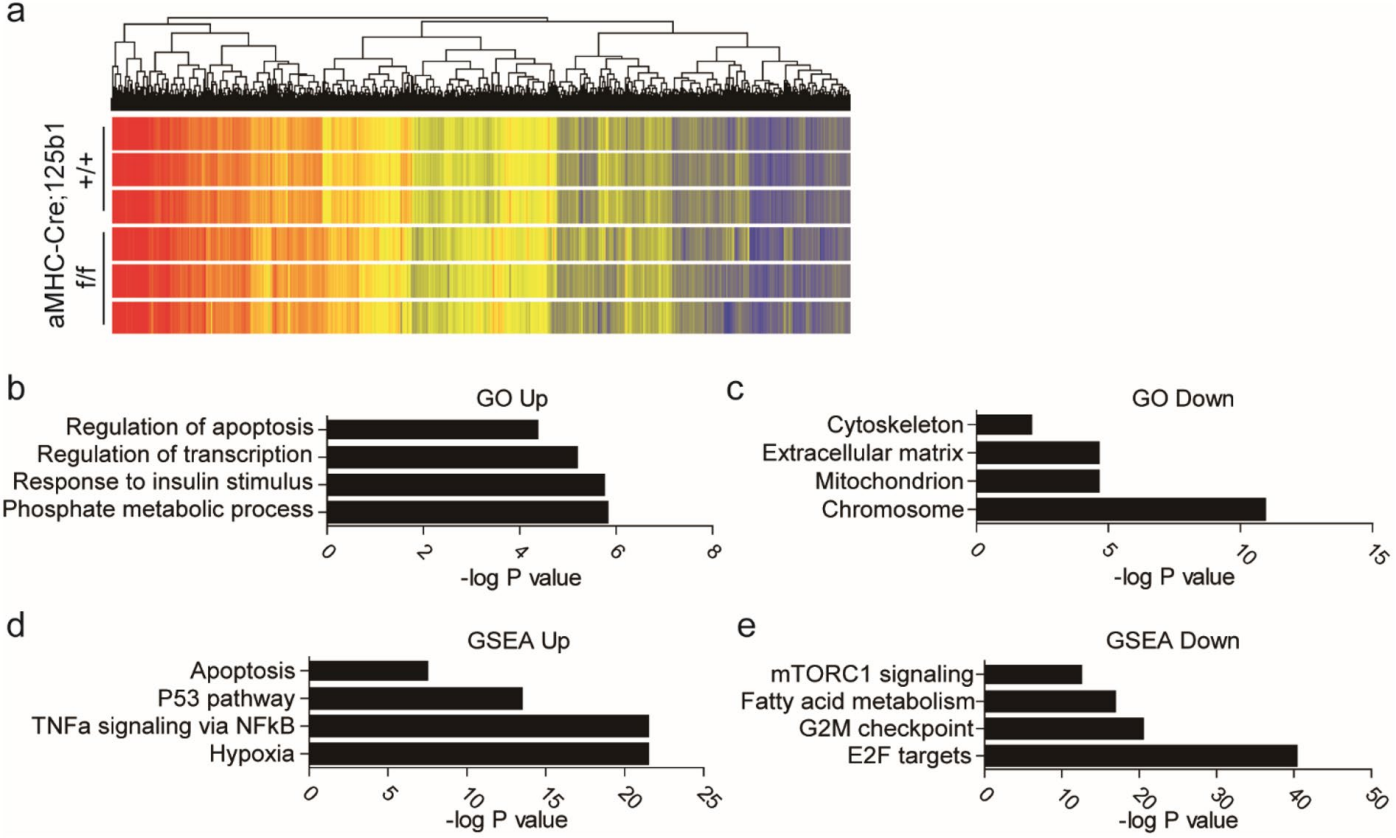
Altered transcriptome in miR-125b deficiency mice. (**a**) Heatmap of microarray. (**b**) Gene ontology of upregulation genes in the knockout neonates. (**c**) Gene ontology of downregulation genes in the knockout neonates. (**d**) Gene set enrichment assay (GSEA) with the Hallmark gene set in the upregulation genes. (**e**) Gene set enrichment assay (GSEA) with the Hallmark gene set in the downregulation genes.

**Figure 6:**
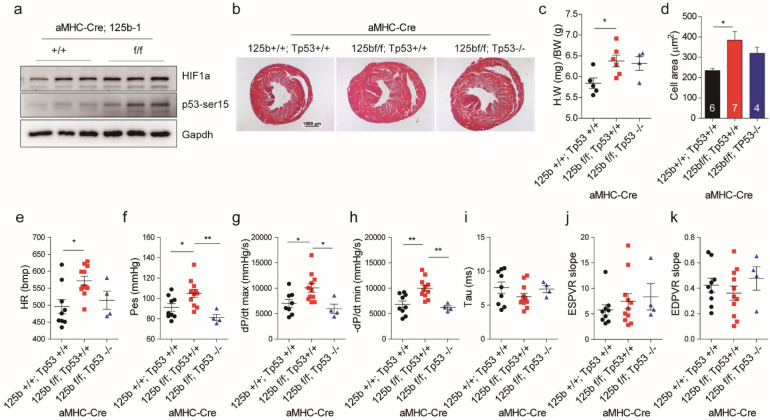
Knockout of Tp53 rescued the increase of heart rates and end systolic pressure in cardiac miR-125b deficient mice, but not cardiac hypertrophy phenotype. (**a**) Expression of HIF1a and p53 ser15phosphorylation in wild-type and miR-125b knockout neonatal hearts. The original images are displayed in Fig. S5. HIF1a, p53 ser15phosphorylation and Gapdh were from different blots with the same protein extracts. (**b**) HE staining of hearts in the control (125b + / + ; Tp53 + / +), miR-125b knockout (125bf/f; Tp53 + / +) and miR-125b/Tp53 double knockout (125bf/f; Tp53−/−) mice. Bar: 1 mm. (**c**) Heart weight-to-body weight ratio in the control, miR-125b knockout and miR-125b/Tp53 double knockout mice (n = 5, 6 and 4. control vs. miR-125b knockout, *p* = 0.0450; control vs. double knockout, *p* = 0.1146; miR-125b knockout vs. double knockout, *p* = 0.9549). (**d**) Cardiomyocyte size in the control, miR-125b knockout and miR-125b/Tp53 double knockout mice (n = 6, 7 and 4. control vs. miR-125b knockout, *p* = 0.0112; control vs. double knockout, *p* = 0.2536; miR-125b knockout vs. double knockout, *p* = 0.4138). (**e**) The heart rate (control vs. miR-125b knockout, *p* = 0.0116; control vs. double knockout, *p* = 0.8295; miR-125b knockout vs. double knockout, *p* = 0.1760), (**f**) end-systolic pressure (control vs. miR-125b knockout, *p* = 0.0363; control vs. double knockout, *p* = 0.3478; miR-125b knockout vs. double knockout, *p* = 0.0055), (**g)** dP/dt maximum (control vs. miR-125b knockout, *p* = 0.0396; control vs. double knockout, *p* = 0.7684; miR-125b knockout vs. double knockout, *p* = 0.0265 ), (**h**) dP/dt minimum (control vs.vs. miR-125b knockout, *p* = 0.0028; control vs. double knockout, *p* = 0.8520; miR-125b knockout vs. double knockout, *p* = 0.0058), (**i**) Tau (control vs. miR-125b knockout, *p* = 0.2908; control vs. double knockout, *p* = 0.9843; miR-125b knockout vs. double knockout, *p* = 0.5825), (**j**) the slope of end-systolic pressure volume relationship (control vs. miR-125b knockout, *p* = 0.6666; control vs. double knockout, *p* = 0.5919; miR-125b knockout vs. double knockout, *p* = 0.9349) and (**k**) the slope of end-diastolic pressure volume relationship (control vs. miR-125b knockout, *p* = 0.7139; control vs. double knockout, *p* = 0.8718; miR-125b knockout vs. double knockout, *p* = 0.5113) in control, miR-125b knockout and miR-125b/Tp53 double knockout mice (n = 9, 12 and 4). The significance of the differences among control, knockout and double knockout groups were determined by one-way ANOVA with Tukey’s multiple comparison test.

To elucidate the underlying mechanism, the neonatal hearts were subjected to transcriptome analysis. The heat map of gene expression showed the differential expression of genes between the knockout and the control hearts (Fig. 5a). Gene ontology analysis showed that the upregulated genes were associated with apoptosis, transcription, insulin stimulus and metabolic process (Fig. 5b) and the downregulated genes were related to cytoskeleton, extracellular matrix, mitochondria and chromosomes (Fig. 5c). This analysis supports our finding about the abnormality of cytoskeleton and mitochondria in the knockout mice. In addition, gene set enrichment analysis (GSEA) was also performed using the Hallmark gene set. The results suggested that the upregulated genes are involved in apoptosis, P53, TNFa signaling, and hypoxia (Fig. 5d) and the downregulated genes are involved in mTORC, fatty acid metabolism, G2M checkpoint and E2F target (Fig. 5e). An increase in phosphor-p53 at serin-15 and HIF1a were observed in the knockout hearts (Fig. 6a), which indicates the activation of p53 and hypoxia signaling. In order to define the target of miR-125b in vivo, we compared this transcriptome analysis, RISC-seq from hearts^32^ and TargetScan^23^ prediction and found that only 23 genes belonged to the three groups (Fig. S4a). Some of them were picked up for further investigation using primary mouse neonatal cardiomyocytes transfected with miR-125b inhibitor. Only two candidate genes, Btg2 and Pafah1b1, were significantly upregulated after miR-125b inhibition in cardiomyocytes (Fig. S4b). Btg2, BTG anti-proliferation factor 2, is a downstream target of p53, regulating the cell cycle through its regulatory role on transcription factors^33^. Btg2 also binds proteins in mRNA deadenylation complexes to facilitate mRNA degradation. Such a function may prevent cardiac hypertrophy induced by adrenergic stimulation by preventing the accumulation of RNA^33^. Pafah1b1, Platelet-activating factor acetylhydrolase IB subunit alpha, is an enzyme subunit regulating the dynamics of the cytoskeleton through motor protein Dynein^34^. Its functions in cardiovascular development and diseases are mostly unknown. We also evaluated the gene expressions of known potential targets: Bak1, Klf13^12^, Tp53 and Traf6^13^ in the neonatal hearts. Unexpectedly, Bak1, Tp53 and Traf6 showed significant downregulation in the miR-125b deficient hearts (Fig. S4c). This controversial result may be due the difference in vitro and in vivo. Other type of cells might express these genes and cell signaling in the tissue microenvironment might be different from that in cell culture.

Since neither of these genes was able to explain the phenotypes that we found in the knockout mice and the potential of multiple targeting of a miRNA, we next searched for possible pathways, instead of single genes, mediating the deficiency of miR-125b knockout. We noticed that Tp53 signaling is known to be involved in apoptosis and cell cycle regulation and cross-talk with HIF1a, NFkb, mTORC, and fatty acid metabolism. The relationship between miR-125b and Tp53 has been discovered in cancers^35^. In addition to Btg2, we observed that another Tp53 target Tp53inp1 showed significant increase in both RNA and protein level in the deficient mice (Fig. S4d and e). Although the RNA level of Tp53 was downregulated (Fig. S4c), the activity of Tp53 was activated (Fig. 6a). We suspect the function of miR-125b might depend on novel function of Tp53. Previous study suggests that cardiac-specific knockout of Tp53 mice are resistant to pressure-overload-induced cardiac hypertrophy and upregulate mitochondria complex gene expression^36^. Based on these clues, we hypothesized that knockout of Tp53 rescues the phenotypes in miR-125b deficiency mice. To test this hypothesis, we generated aMyh6-Cre; miR-125b-1 f./f; Tp53 −/− double knockout mice. However, the double knockout mice did not rescue perinatal death or cardiac hypertrophy in adult mice. In six-month old mice, both miR-125b and miR-125b/Tp53 knockout presented cardiac hypertrophy determined by tissue morphology (Fig. 6b), heart weight-to-body weight ratio (Fig. 6c) and cardiomyocyte size (Fig. 6d). To further investigate the heart function of the double knockout mice, we measured the cardiac hemodynamics by cardiac catheterization. Interestingly, the miR-125b/Tp53 double knockout mice with slightly reduced heart rate (Fig. 6e) significantly rescued LV systolic pressure and both max dP/dt and min dP/dt (Fig. 6f–h). This suggests that although Tp53 might not be the major cause of neonatal death and adult cardiac hypertrophy in the knockout mice, it still plays a role in the regulation of heartbeats and LV systolic pressure. No difference was found in the relaxation time constant (Tau), end-systolic PV relation slope (ESPVR slope) and end-diastolic PV relation slope (EDPVR slope) in the double knockout mice (Fig. 6i–k).

MicroRNA plays a negative role in protein translation. Therefore, we launched a protein quantification assay by tandem mass tag (TMT) labeling and LC/MS. Unfortunately, none of the upregulated protein was the potential target of miR-125b. The knockout neonatal hearts have more proteins related to chromosome segregation, mitotic nuclear division, cell cycle and cell division (Fig. S4f) and fewer proteins which are associated with mitochondria, the oxidation–reduction process, the fatty acid metabolic process, and fatty acid transport (Fig. S4g). In line with proteomic analysis, most fatty acid entry-related genes were downregulated in the knockout neonatal hearts (Fig. S4h). In both transcriptome and proteome analysis, we noticed the downregulation of fatty acid metabolism was significant. Nine candidate genes were downregulated at both the transcription and protein level mediating fatty acid transport or long-chain fatty acid metabolic processes, including Acadvl, Fabp4, CD36, Hmgcs2, Slc27a1, Acot1, Acot2, Plin5, and Hsdl2 (Fig. S4i, j). Except for Fabp4, eight other candidate genes are located in the mitochondrion. These data suggested a deficiency in mitochondria and fatty acid metabolism may be the cause of the phenotype in the knockout mice. The dysregulation of fatty acid metabolism may cause accumulation of excess lipids in cardiomyocytes. Therefore, we examined the free fatty acid amount in the neonatal hearts and found more fatty acid in the knockout mice than in control mice (Fig. S4k). These data indicate that miR-125b may be essential for energy homeostasis, especially from fatty acid metabolism, in the hearts.

## Discussion

In the case of cardiac-specific Dicer knockout, miR-125b is one of the significantly downregulated miRNAs^8^. Other downregulated miRNAs include miR-1, miR-208, miR-499, and miR-133^8^. However, most of the targeted deletion of miRNAs in the heart showed no obvious phenotypes^37–39^, except miR-1^40^. The knockout of miR-125b-1 causes perinatal death and induced cardiac hypertrophy in adulthood, which means miRNA-125b is crucial.

The expression level of miR-125b changes under different cardiac pathological status. It is induced by angiotensin II in cardiac fibroblasts^41^ and upregulated in human failing hearts^29^. However, it is downregulated after myocardial infarction (Fig. S5) and under ischemia/reperfusion injury^13^. It is possible that the regulatory role of miR-125b in different cardiac diseases is through multiple mechanisms and cell types. Previous studies have demonstrated that miR-125b protects cardiomyocytes from injury induced apoptosis^12,13^ through suppression of Tp53 signaling^12,13^. We performed TUNEL staining in the neonatal hearts and found that the numbers of apoptotic cardiomyocytes in both the control and knockout neonatal hearts were small (Fig. S6a). The apoptosis-related Tp53 downstream targets Noxa and Puma showed no difference in expression (Fig. S6b). Apoptosis is unlikely the cause of perinatal death in our deficient mice. Another function of miR-125b in hearts is to promote fibroblast-to-myofibroblast transition^14^. We performed Trichrome staining to evaluate cardiac fibrosis in hearts, but we barely observed any staining in neonatal and young adult hearts (Fig. S6c, e). Therefore, fibrosis-related genes including Acta2, Col1a1, Postn, Tgfbr1 and Vim were analyzed. Interestingly, Acta2, Col1a1 and Tgfbr1 were significantly downregulated in miR-125b deficient neonatal hearts (Fig. S6d) but Tgfbr1 and Vim were significantly upregulated in young adult hearts (Fig. S6f). The cardiac fibrosis became obvious in mature adult hearts and quantification of the fibrotic area by Trichrome staining displayed a significant increase (Fig. S6g). These data imply that the cardiac specific knockout of miR-125b-1 had the potential to suppress fibrosis at the neonatal stage. However, the fibrosis occurring in adulthood could be the consequence of cardiomyopathy in the deficient mice. In agreement with previous studies, we found miR-125b is a protective microRNA in cardiomyocyts^12,13^, but different from the cardiac infarction model, anti-apoptosis might not be the major role of miR-125b during development. In fibroblasts, overexpression of miR-125b triggers fibroblasts activation^14^. Interestingly, the cardiac specific miR-125b-1 knockout downregulated fibrosis-related gene expression at the neonatal stage, which indicates that miR-125b in cardiomyocytes may be able to regulate cardiac fibrosis.

Our transcriptomic and proteomic analysis revealed that fatty acid metabolism has the key role in cardiac miR-125b deficient mice. Fatty acids are predominant sources of ATP production in hearts and a recent quantification study also directly proved this concept^42^. The exquisite balance between fatty acid uptake and oxidation keeps the heart healthy. In all kinds of cardiac diseases, alteration of the balance often leads to lipid accumulation in cardiomyocytes and causes cardiac lipotoxicity^43^. For example, impairment of fatty acid uptake was found in patients with dilated cardiomyopathy^44^. In addition, myocardial fatty acid metabolism was a prognostic marker for cardiac hypertrophy in hypertension patients^45^. Studies in genetically manipulated mice also demonstrated that lipid accumulation alone causes heart failure^46^. ROS generation, defective insulin signaling, apoptosis and ER stress are potential mechanisms explaining the cardiomyopathy caused by lipid metabolism^43^. However, exactly how miR-125b regulates fatty acid metabolism remains unclear. Notwithstanding, this study reports a novel link between microRNA-125b and fatty acid metabolism in hearts and further investigation is required.

The mtDNA copy number has been considered to be an indicator of mitochondrial biogenesis and often linked to ATP production^47^. However, in miR-125b deficient neonatal hearts, we observed an increased mitochondrial DNA copy number (mtDNA) with lower ATP production. In our case, the mtDNA copy number was not related to energy production. A study reported that mtDNA copy number can be induced by oxidative stress and free radicals^48^. For example, the sub-lethal concentrations of antimycin A, a mitochondrial electron transport chain complex III inhibitor, and non-lethal concentrations of H_2_O_2_ induced mtDNA copy number in human cells^49^. This induction is thought to compensate for the defective mitochondria under tolerable stress and which may be related to cell-cycle arrest^49^, since mtDNA replication is mainly at the S/G2 phases^50^. In our transcriptomic analysis, we noticed miR-125b deficiency influenced cell cycle regulation (Fig. 5e). It is possible that circulatory system switch from fetus to newborns endures certain ROS stress^51^. The miR-125b deficient mice were vulnerable to such stress and induce more mitochondrial DNA replication. ROS can impair mtDNA-encoded transcription and mitochondrial oxidative phosphorylation protein levels^52^, which is similar to what we have observed in miR-125b deficient mice. We suspected that moderate ROS stress may perturb mitochondrial biogenesis and integrity. Mitophagy is an important cellular mechanism to maintain the quality and the quantity of mitochondria by removing damaged mitochondria and it requires mitochondria fusion and fission^53–56^. Although the upstream trigger is not known, the activation of mitochondrial fission, fusion and mitophagy in the miR-125b deficient mice may be due to the cellular mechanism of mitochondria homeostasis in cardiomyocytes.

There are several methods to determine heart failure with preserved ejection fraction (HFpEF). The abnormal E/A ratio was observed in neonatal hearts (Fig. 1t, u) not in the adult mice. Instead, we observed the decrease of the ratio of early diastolic mitral inflow velocity to early diastolic septal mitral annulus velocity (E/E' ratio) in mature adult knockout mice (Fig. 3m, n). However, the hemodynamic assessment from cardiac catheterization did not show the difference in EDPVR (Fig. 6k). In addition, the heart rate and the end-systolic pressure may influence the readout of cardiac function^18^. Because of the stages of diastolic dysfunction and the limitations of the techniques, several assessments may be required to determine HFpEF to provide early treatment and diagnosis.

We generated cardiac-specific miR-125 knockout to uncover its physiological role in hearts. However, the generation of these knockout mice raised more questions. The perinatal death and cardiac hypertrophy phenotype were evident but the mechanisms behind the phenotype are intriguing. Both transcriptome and proteasome analysis points to a defect in fatty acid metabolism, but the potential targets of miR-125b were undefined. The unexplored role of miR-125b, especially mito-miR-125b, will be further investigated to link the phenotype and the deficiency of miR-125b in hearts.

The cardiac specific miR-125b-1 knockout mice showed perinatal death and cardiac deficiency in mature adulthood. The insufficiency of miR-125b in neonatal stage influenced mitochondrial function, biogenesis and dynamics. In addition, both transcriptome and proteome approaches pointed out the dysregulation of fatty acid metabolism in the knockout mice, which is particularly crucial for cardiac energy homeostasis. In conclusion, this is the first study demonstrate that microRNA-125b regulates cardiac metabolism and revels the physiological role of miR-125b in heart.

## Methods

### Construction of Mir125b1 targeting vector and generation of Mir125b1 conditional KO mice

A targeting vector was constructed using a recombineering approach previously developed by Dr. Copeland’s group^57,58^. A BAC clone (clone no. bMQ-48P22, Geneservice) from a 129S7/AB2.2 BAC library containing the mouse *Mir125b1* genomic region was used. The vector was designed to conventionally and conditionally delete the *Mir125b1*. At the first, a nearly 16.7 kb genomic region was retrieved from the BAC clone into the pL253 vector^59^ carrying the HSV-tk for negative selection. The pL253 carrying the *Mir125b1* genomic DNA (pL253-*Mir125b1*) was further engineered to insert a 34-bp *loxP* sequences at the upstream of *Mir125b1* and a 1.9-kb *frt*-*neo*-*frt*-*loxP* cassette (*neo* cassette, derived from pL451^59^) at the downstream of *Mir125b1* for positive selection. The targeting vector was linearized by *Not*I restriction enzyme and electroporated into R1 embryonic stem (ES) cells. After double selection by G418 (240 μg/ml) and Ganciclovir (2 μM) antibiotics, those double-resistant ES cell clones were further analyzed by Southern blot to select correct clones. For conditional KO of *Mir125b1*, the ES cell clones harboring the loxP flanked region were transiently transfected with a vector expressing the Flp recombinase (PCAGGS-PTD-NLS-Plp) to delete the neo cassette. Correct ES clones were identified by PCR screening and then injected into the blastocysts derived from C57BL/6 donors to produce chimeras. After breeding with wild-type C57BL/6 mice, the germ line transmission of the floxed allele into offspring was confirmed by PCR strategy.

### Animal studies

The miR-125b-1flox/flox allele was generated by flanking the miR-125b-1 in this cluster with loxP sites using standard homologous recombination in ES cells. Generation and genotyping of the αMHC-Cre line has been described previously^60^. Genotyping primers for these alleles are listed in Table S1. All animal procedures were approved by the Experimental Animal Committee, Academia Sinica, Taiwan, and carried out in accordance with their guidelines and the ARRIVE guidelines. For the treatment of isoproterenol, osmotic pumps were implanted surgically and subcutaneously for 14 days at a dose of 60 mg/kg/day.

### Echocardiography

Isoflurane inhalation was used for the in vivo measurement of echocardiography. The echocardiography was performed again to measure the left ventricle end-diastolic and end-systolic volumes (LVEDV and LVESV, respectively). The left ventricular ejection fraction (LVEF, %) was calculated as (LVEDV-LVESV)/LVEDV × 100%. For measurement of ejection fraction in neonates, Doppler echocardiography was performed with Vevo 3100 (Fujifilm).

### Histology staining

All heart tissues were fixed by 4% paraformaldehyde overnight. To apply HE staining, paraffin embedded tissues underwent deparaffinization and were stained with Mayer’s hematoxylin and eosin. Collagen deposition was visualized using Trichrome Stain Kit (Sigma) according to the manufacturer’s protocol.

### Real-time quantitative polymerase chain reaction

RNA was extracted from hearts using Trizol (Invitrogen) and reverse-transcribed using SuperScript III Reverse Transcriptase (Invitrogen). Real-time PCR was performed using an ABI 7500 real time PCR system (Applied Biosystems). The primers are listed in Table S2. For miRNA study, isolated RNAs were reversed-transcribed using the Taqman primer sets for miRNAs (Ambion) with MultiScribe Reverse Transcriptase (Applied Biosytems). Real-time PCR was conducted with the Taqman 2 × Universal Master Mix (Applied Biosystems).

### Immunofluorescence microscopy

Heart tissue sections were deparaffinized and rehydrated before staining. Wheat germ agglutinin staining was performed using FITC conjugated-wheat germ agglutinin (Invitrogen) for 10 min at room temperature.

### Transmission electron microscopy

Following systemic perfusion, the heart was quickly removed and washed. The small pieces of tissues were pre-fixed in 4% paraformaldehyde plus 2% glutaraldehyde in PBS overnight at 4 °C. Following that, the tissues were washed and post-fixed in 1% OsO_4_ for one hour on ice. After washing, the tissues were stained with 2% uranyl acetate for 40 min on ice. The tissues were washed using distilled water, dehydrated in upgraded ethanol series and polymerized in the Spurr embedding medium overnight at 60 °C. Imaging was performed using a Tecnai G2 Spirit TWIN transmission electron microscope.

### Quantification of mitochondrial number

Genomic DNA was extracted from neonatal ventricles. The tissue was lysed with 250 µl of lysis buffer (50 mM Tris pH8; 100 mM EDTA; 0.5% SDS) with 200 µg/ml proteinase K at 55 °C for 6 h and then 80 µl of 5 M potassium acetate was added to precipitate proteins. After centrifuge at 13,500 rpm, 4 °C for 30 min, the supernatant was transferred into a new tube. Genomic DNA was precipitated with absolute EtOH and washed with 70% EtOH. After drying, the pellets were resolved in TE buffer with a concentration of 200 ng/µl. According to previous study^61^, the primers used for detection of nuclear genomic DNA were: mB2MF1:ATGGGAAGCCGAACATACTG and mB2MR1: CAGTCTCAGTGGGGGTGAAT. The primers used for detection of mitochondrial genomic DNA were: mMitoF1: CTAGAAACCCCGAAACCAAA and mMitoR1: CCAGCTATCACCAAGCTCGT.

The ratio of mitochondrial genomic DNA and nuclear genomic DNA was determined by real-time PCR.

### ATP assay

ATP concentration in the neonatal hearts was determined by ATP colorimetric/fluorometric assay kit (BioVision), following the instructions. One neonatal heart was homogenized in 100 µl ATP assay buffer. Protein was removed by 10 kDa spin-column. A total 50 µl of lysate was used for the measurement of ATP concentration by OD570 nm.

### Mitochondria isolation

Mitochondria were isolated from minced mouse hearts or cells and were homogenized with a glass tissue grinder using buffer containing 1 M sucrose, 0.1 M EGTA/Tris and 1 M Tris/HCl. The homogenate was further centrifuged at 600 g for 10 min at 4 °C. The supernatants were centrifuged at 7000 g for 10 min at 4 °C and the resulting supernatants were discarded. The pellet containing the mitochondria was washed and centrifuged at 7000 g for 10 min at 4 °C three times before resuspension. The quality of mitochondria isolation was confirmed by western blot using cytosol protein alpha-tubulin and mitochondrial protein Vdac2 and Cox4 (Fig. 4a).

### Western blotting

Mitochondrial proteins or total proteins were collected from either whole heart or cells using lysis buffer. The antibodies used were anti-PINK1, anti-LC3b, anti-VDAC2, anti CoxIV, anti-p62, anti Trp53inp1 (GeneTex), anti-OXPHOS cocktail (Abcam) and anti-GAPDH (Millipore). Western blot images were captured by BioSpectrum Auto Imaging System with UVP Vision WorksLS software. In general, the acquisition setting is a time-lapse condition: 10 images within 2mins for high intensity signal or 20 images within 5mins for low intensity signal.

### Cardiac catheterization

Mice were anesthetized by i.p injection of urethane (1 g/kg) followed by s.c. injection of 0.5 ml normal saline and buprenorphine 0.1 mg/kg. Mice were intubated with a soft tube which was connected to a ventilator, then a SPR-839 catheter was inserted into the left ventricle through the right carotid artery. Pressure and volume in the LV chamber were recorded by a Millar MVPS system and analyzed by Powerlab software.

### Gene expression microarray

Total RNA was isolated from the ventricles using Trizol (Invitrogen) according to the manufacturer’s protocol and purifed with RNeasy Mini Kit (Qiagen). Microarray analysis was performed employing Affymetrix GeneChip assays using three independent experiments by the Affymetrix Gene Expression Service Lab (http://ipmb.sinica.edu.tw/affy/) supported by Academia Sinica, Taiwan using the Mouse Genome 430 2.0 Array. Differential expressions of genes were analyzed by GeneSpring GX software version 11.5 (Agilent Technologies) with Log2FC cutoff = 1, and a *P* value cutoff < 0.05. All data were submitted to GEO. Accession no. GSE157746.

### Proteomic analysis

The proteomics assays were supported by the IBMS Proteomics Core Facility, Academia Sinica. Ten aliquots of tissue lysate (100 µg each) were reduced by 100 mM of DTT, alkylated by 50 mM of iodoacetamide and digested by trypsin. Isobaric labeling of the peptides was performed using 10-plex tandem mass tag (TMT) reagents (Thermo Scientific). TMT reagents were added to 100 μg of peptides for labeling. Labeled peptides from different samples were combined and desalted before fractionation using an XBridge BEH130 C18 column (3.5 μm 2.1 × 150 mm, Waters, MA, USA) on a 1100 series HPLC (Agilent). A total of 80 × 1 min fractions (200 μL each) were collected. The 15 pooled fractions were lyophilized and then subjected to LC–MS/MS analysis. It was performed on a nanoACQUITY UPLC System (Waters, USA) coupled to a high resolution mass spectrometer Q Exactive HF-X instruments (Thermo Fisher Scientific, Bremen, Germany). The raw spectra were processed using Proteome Discoverer v.2.2 (Thermo Fisher Scientific, Waltham, MA, USA). The MS/MS spectra were searched with the Mascot engine against the UniProtKB/Swiss-Prot human database.

### Free fatty acid quantification

Neonatal ventricles were homogenized with 1% Triton X-100 in chloroform. The total lipids were at the organic phase, separated by centrifugation at 12,000 × g for 15 min. Total lipids were air-dried at 50 °C until pellets formed and then were subjected to vacuum to remove trace chloroform. The amount of free fatty acid was determined using a free fatty acid quantification colorimetric/fluorometric kit (BioVision).

### Gene ontology and Gene set enrichment analysis

Gene ontology was performed by DAVID Bioinformatics Resources 6.8^62,63^. The Gene set enrichment analysis was performed by GSEA^64,65^ with the Hallmark gene set.

### Data analysis

All data are expressed as the mean ± SEM. Statistical significance was determined using a 2-tailed Student’s t test or ANOVA, as appropriate. Differences between groups were considered statistically significant at P values of less than 0.05. All statistical analysis and graphs were performed using GraphPad Prism 8.

## Supplementary Information

Supplementary Information.

## Data Availability

The data, analytical methods, and study materials for the purposes of reproducing the results or replicating procedures can be made available on request to the corresponding author.
