# Supplementary material for: Cardiac-specific microRNA-125b deficiency induces perinatal death and cardiac hypertrophy
Source: Sci Rep. 2021 Jan 27;11:2377. doi: 10.1038/s41598-021-81700-y (PMC7840921; doi:10.1038/s41598-021-81700-y)
Supplement: Supplementary file 1 — Supplementary Information. [file 41598_2021_81700_MOESM1_ESM.pdf]

Supplementary information

Cardiac-specific MicroRNA-125b deficiency induces perinatal death and cardiac hypertrophy

Chen-Yun Chen[1,3], Desy S. Lee[1], Oi Kuan Choong[1], Sheng-Kai Chang[2], Tien Hsu[3], Martin W. Nicholson[1], Li-Wei Liu[1], Po-Ju Lin[1], Shu-Chian Ruan[1], Shu-Wha Lin[2], Chung-Yi Hu[2], Patrick C.H. Hsieh[1, 4-5]*

[1]Institute of Biomedical Science, Academia Sinica, Taipei 115, Taiwan
[2]Department of Clinical Laboratory Sciences and Medical Biotechnology, College of Medicine, National Taiwan University, Taipei 100, Taiwan
[3]Department of Biomedical Sciences and Engineering, National Central University, Taoyuan 320, Taiwan
[4]Institute of Medical Genomics and Proteomics, National Taiwan University College of Medicine, Taipei 100, Taiwan
[5]Institute of Clinical Medicine, National Taiwan University College of Medicine, Taipei 100, Taiwan

Supplementary: 11 figures, 2 tables

Figure S1

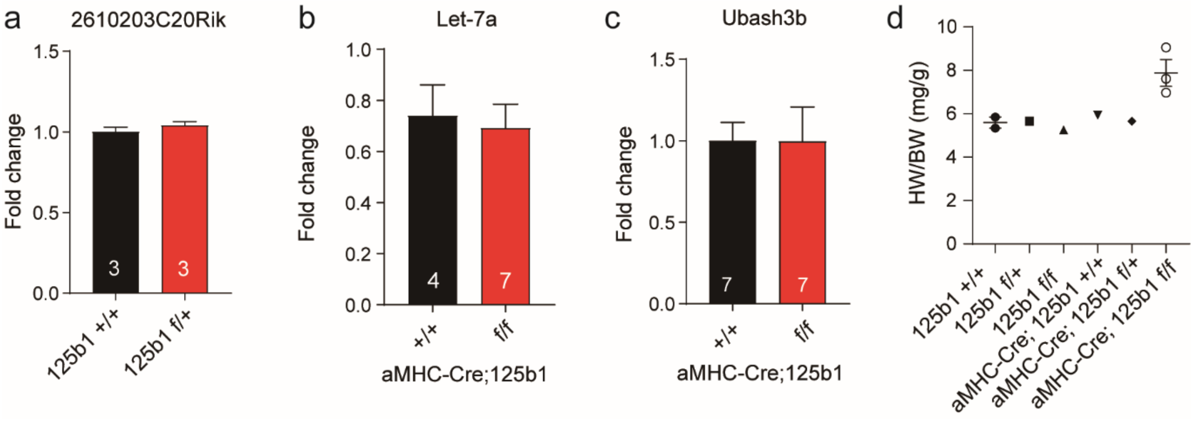

Figure S1. The effect of the loxP insertion on host and neighbor gene expression and mouse phenotypes. (**a**) The expression of 2610203C20Rik in miR-125b-1 +/+ and miR-125b-1 f/+ littermates. RNAs were extracted from 2-week-old mouse hearts. (n=3 for each group, *p*=0.3284 for Student *t* test). (**b**) Let-7a expression in control and miR-125b-1 knockout neonatal hearts. (n=4 vs. 7, *p*=0.7623 for Student *t* test). (**c**) Ubash3b expression in control and miR-125b-1 knockout neonatal hearts. (n=7 for each group, *p*=0.9889 for Student *t* test). (**d**) Heart weight-to-body weight ratio from 6-month-old adult littermates.

Figure S2

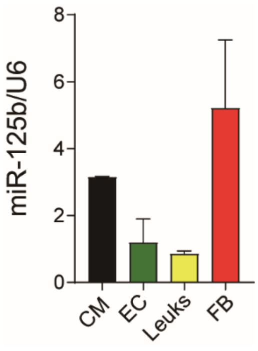

Figure S2. MiR-125b expression in different cells types from adult hearts. CM: cardiomyocyte; EC: endothelial cell; Leuks: leukocytes; FB: fibroblasts (n=2).

Figure S3

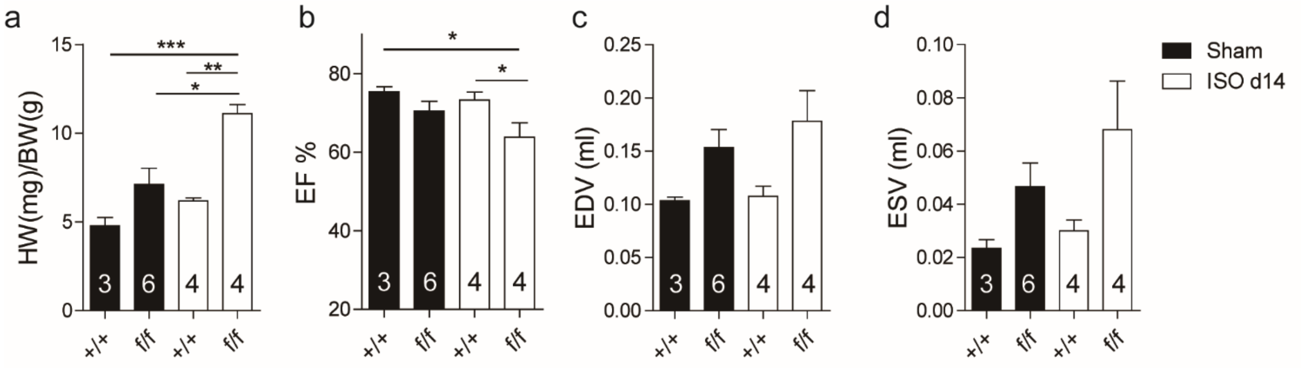

Figure S3. Cardiac-specific miR-125b knockout mice were more susceptible to treatment with isoproterenol. (**a**) The ratio of heart weight-to-body weight in the control and knockout mice with or without isoproterenol treatment (n=3, 6, 4 and 4 in control without treatment, knockout without treatment, control with ISO treatment and knockout with ISO treatment group, control sham vs. knockout sham, *p*=0.1653; control sham vs. control ISO, *p*=0.6803; control sham vs. knockout ISO, *p*=0.0008; knockout sham vs. control ISO, *p*=0.7694; knockout sham vs. knockout ISO, *p*= 0.0121; control sham vs. knockout ISO, *p*=0.0044). (**b**) The ejection fraction of the control and knockout mice with or without isoproterenol treatment (n=3, 6, 4 and 4 in control without treatment, knockout without treatment, control with ISO treatment and knockout with ISO treatment group, control sham vs. knockout sham, *p*=0.5914; control sham vs. control ISO, *p*=0.9550; control sham vs. knockout ISO, *p*=0.0217; knockout sham vs. control ISO, *p*=0.8550; knockout sham vs. knockout ISO, *p*= 0.0872; control sham vs. knockout ISO, *p*=0.0361). (**c**) The end-diastolic volume (n=3, 6, 4 and 4 in control without treatment, knockout without treatment, control with ISO treatment and knockout with ISO treatment group, control sham vs. knockout sham, *p*=0.3946; control sham vs. control ISO, *p*=0.9993; control sham vs. knockout ISO, *p*=0.1321; knockout sham vs. control ISO, *p*=0.3911; knockout sham vs. knockout ISO, *p*= 0.7849; control sham vs. knockout ISO, *p*=0.1182) and (**d**) the end-systolic volume of the control and knockout mice with or without isoproterenol treatment (n=3, 6, 4 and 4 in control without treatment, knockout without treatment, control with ISO treatment and knockout with ISO treatment group, control sham vs. knockout sham, *p*=0.5885; control sham vs. control ISO, *p*=0.9860; control sham vs. knockout ISO, *p*=0.1279; knockout sham vs. control ISO, *p*=0.7503; knockout sham vs. knockout ISO, *p*= 0.5378; control sham vs. knockout ISO, *p*=0.1710).

Figure S4

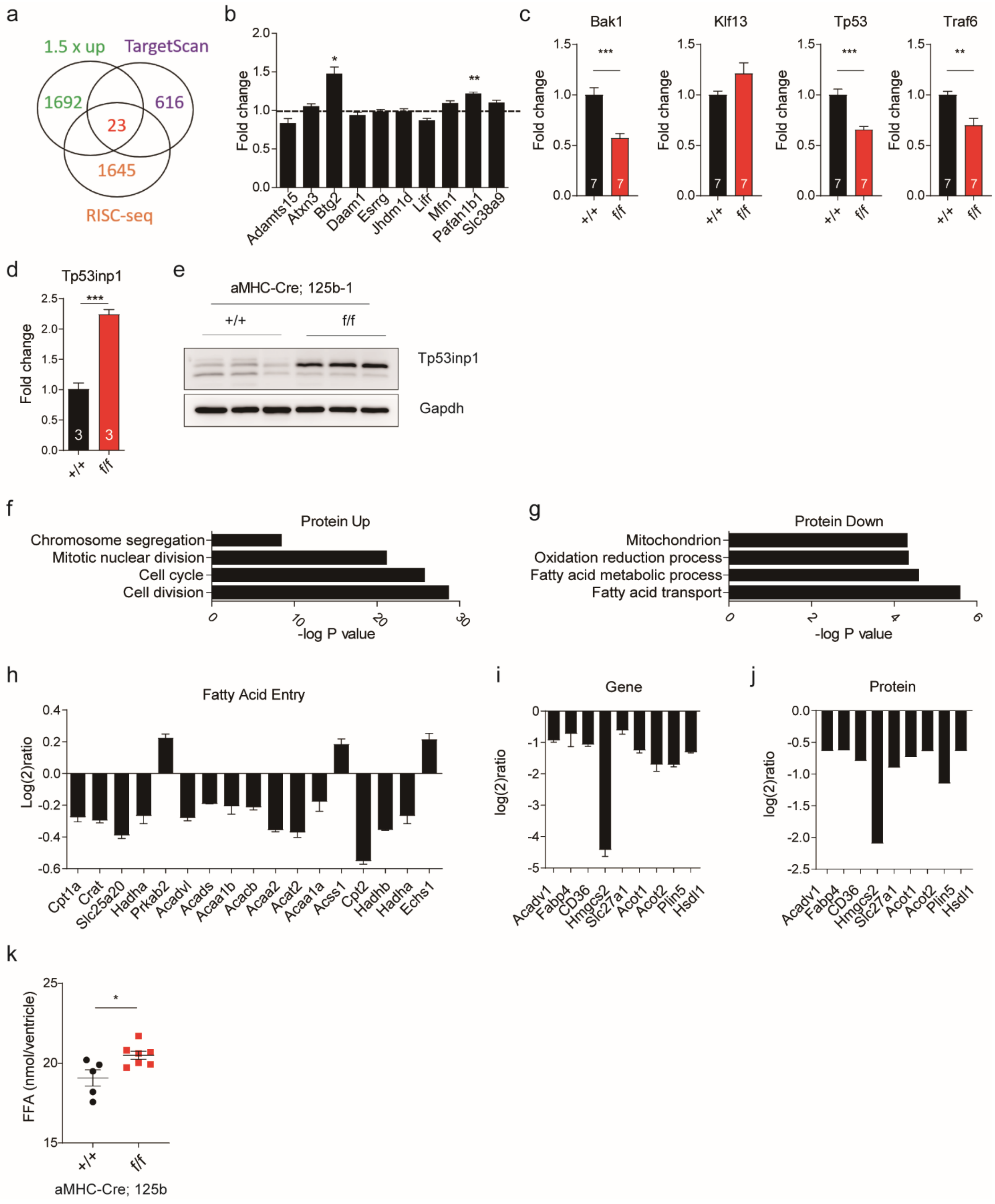

Figure S4. Identification of miR-125b downstream target by transcriptome and proteome analysis. (**a**) Identification of potential target genes by Venn diagram. An upregulated database, a heart RISC-seq database and TargetScan 6.0 prediction were used to identify candidate genes. (**b**) Validation of common candidate gene expression after LNA-125b transfection in mouse neonatal cardiomyocytes. The result was from four independent experiments. (**c**) Bak1, Klf13, Tp53 and Traf6 expression in

control and miR-125b deficient ventricles. (n=7 for each group, Bak1 p=0.0003, Klf13 p=0.0802, Tp53 p=0.0002, Traf6 p=0.0021 for unpaired Student t test) (**d**) Tp53inp1 expression in control and miR-125b deficient ventricles. (n=3 for each group, p=0.0006 for unpaired Student t test) (**e**) Western blot of Tp53inp1 in neonatal hearts. (**f**) GO analysis of upregulated proteins. (**g**) GO analysis of downregulated proteins in the knockout neonatal hearts. (**h**) The expression changes of fatty acid related genes, retrieved from microarray analysis, between miR-125b deficient and control hearts. (**i**) Expression change of nine candidate genes. Bar and error bar indicate mean and SEM; n=3. (**j**) Expression change of nine candidate proteins. Bar indicates mean; n=3. (**k**) Free fatty acid amount in the neonatal hearts (n=5 vs. 7, p=0.0209 for unpaired Student t test).

Figure S5

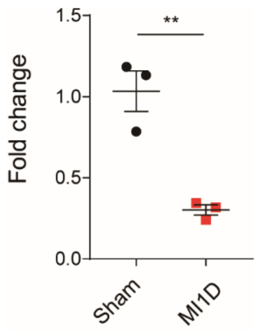

Figure S5. MiR-125b expression after myocardial infarction. The expression level of miR-125b in mouse hearts one day after LAD ligation. (n=3, p=0.0047 for unpaired Student t test)

Figure S6

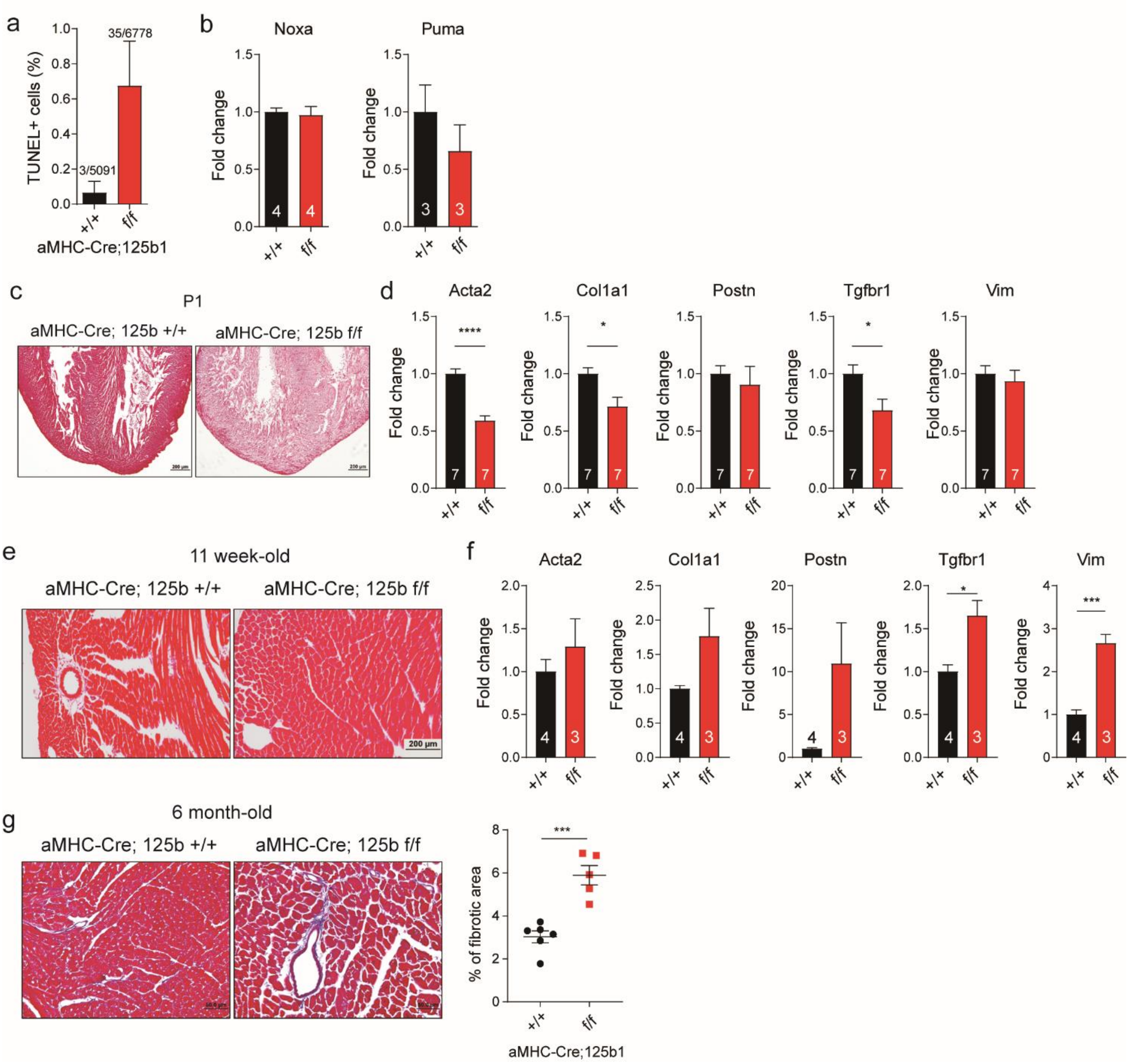

Figure S6. Cardiomyocyte apoptosis and fibrosis in miR-125b deficient mice. (**a**) TUNEL staining in neonatal hearts. Only 3 TUNEL positive cells out of a total 5,091 cardiomyocytes found in control neonatal hearts and 35 TUNEL positive cells out of a total 6,778 cardiomyocytes found in miR-125b deficient neonatal hearts (n=3 vs.. 4, p=0.1045 for unpaired Student t test). (**b**) Expression of Noxa and Puma in miR-125b deficient neonatal hearts (Noxa: n=4 for each group, p=0.7411; Puma: n=3 for each group, p=0.3568 for unpaired Student t test). (**c**) Trichrome staining of neonatal hearts. (**d**) Acta2, Col1a1, Postn, Tgfbr1 and Vim expression in neonatal hearts (n=7 for each group, Acta2 p<0.0001; Col1a1 p=0.0126; Postn p=0.5953; Tgfbr1 p=0.0254; Vim p=0.5468 for unpaired Student t test) (**e**) Trichrome staining of 11-week-old mouse hearts. (**f**) Acta2, Col1a1, Postn, Tgfbr1 and Vim expression in 11-week-old mouse hearts. (n=4 vs. 3, Acta2 p=0.4077; Col1a1 p=0.6371; Postn p=0.0552; Tgfbr1 p=0.0145; Vim p=0.0006 for unpaired Student t test) (**g**) Trichrome staining of 6-month-old mouse hearts. The fibrotic area was measured by ImageJ. (n=6 vs. 5, p=0.0003 for unpaired Student t test).

Figure S7

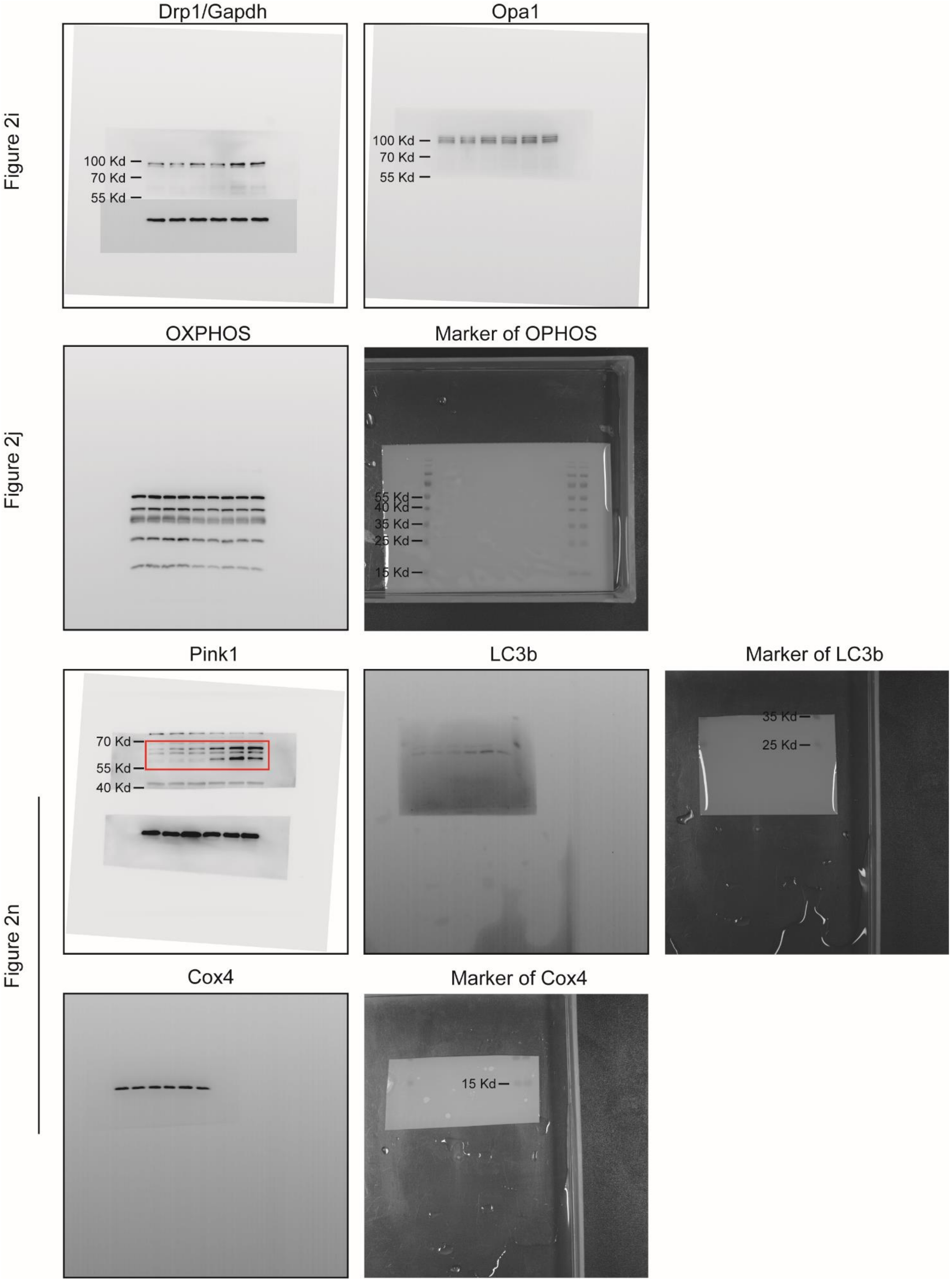

Figure S7. The original images of western blot for Figure 2.

Figure S8

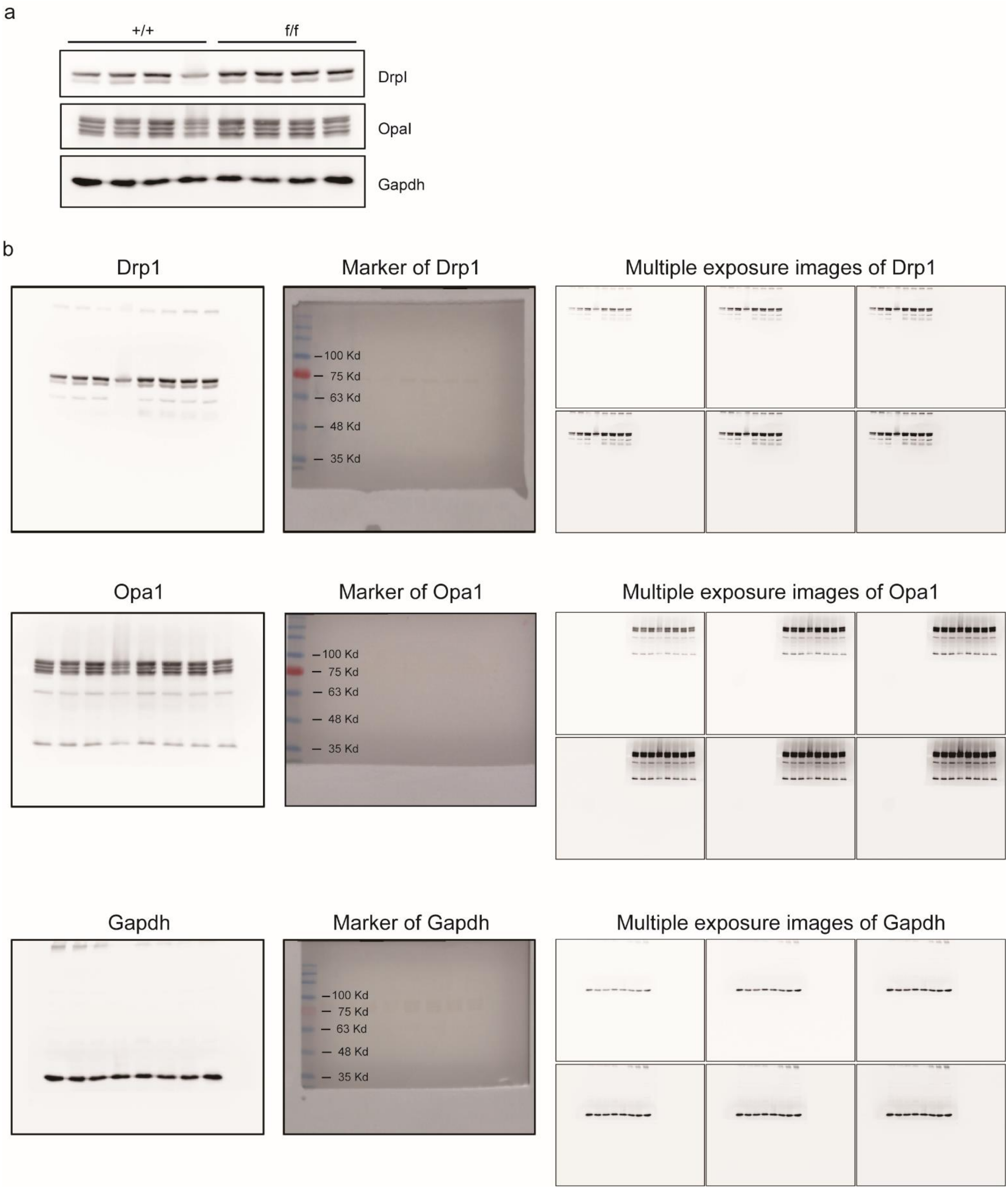

Figure S8. Repeat experiment as Figure 2i. (**a**)Western blot of Drp1, Opa1 and Gapdh. Opa1 and Gapdh were from the same blot. Drp1 was from the other blot with the same protein extracts. (**b**) the original images of western blot.

Figure S9

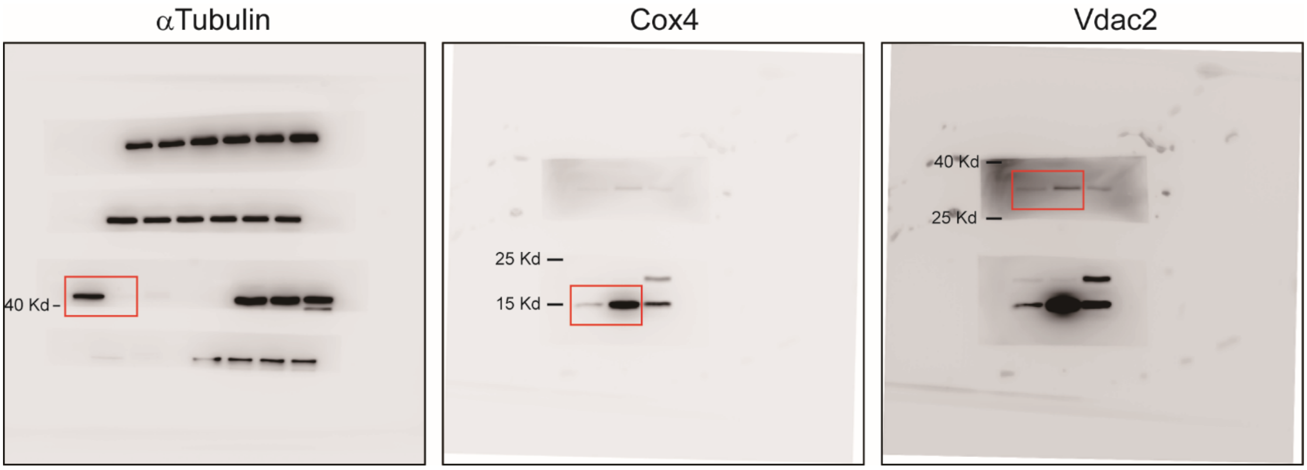

Figure S9. The original images of western blot for Figure 4.

Figure S10

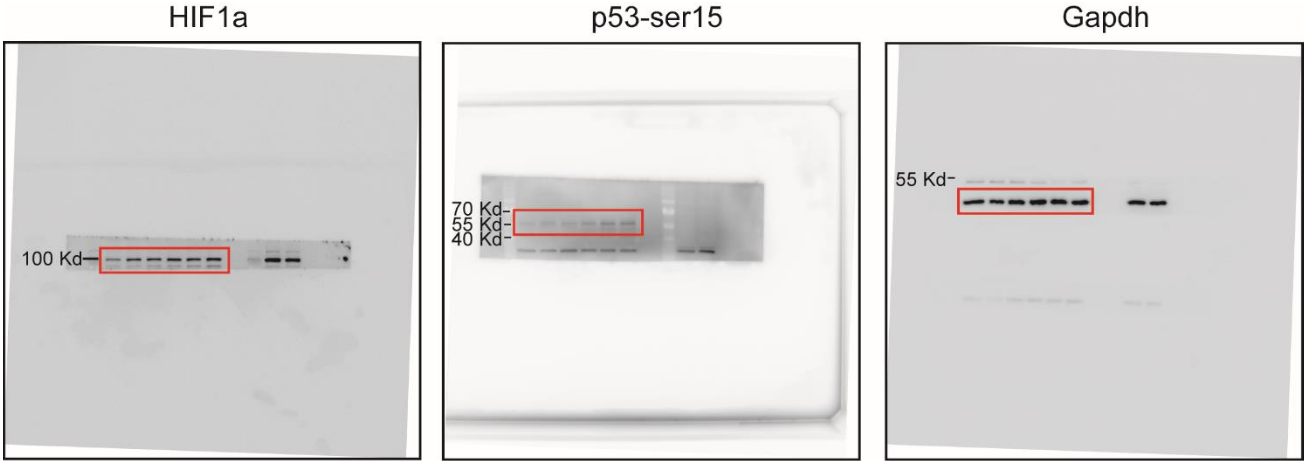

Figure S10. The original images of western blot for Figure 6.

Figure S11

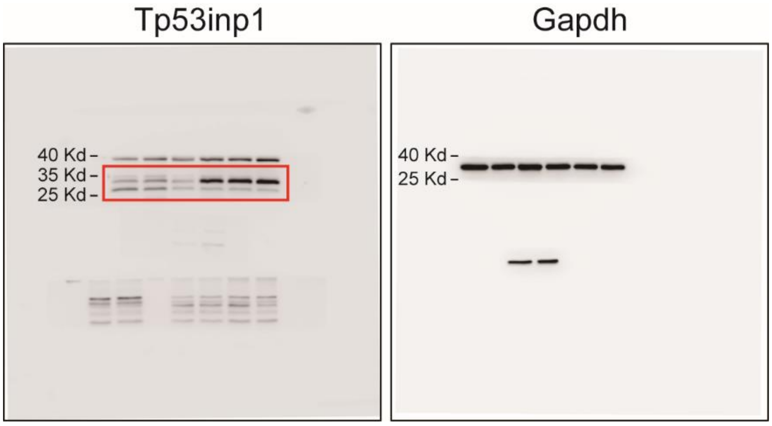

Figure S11. The original images of western blot for Figure S4e.

Table S1. Genotyping primer list

Name	Forward	Reverse
aMHC-Cre	GCG GTC TGG CAG TAA AAA CTA TC	GTG AAA CAG CAT TGC TGT CAC TT
miR-125b1	GTT ACA CAC TTC TCA GTT CAG CG	GGA GTG AAA GAA ATA GCT GGG TG
Tp53	mu: CAG CCT CTG TTC CAC ATA CAC T<br>wt: AGG CTT AGA GGT GCA AGC TG	TGG ATG GTG GTA TAC TCA GAG C

Table S2. qPCR primer list

Name	Forward	Reverse
bMHC	GTG CCA AGG GCC TGA ATG AG	GCA AAG GCT CCA GGT CTG A
ANP	CGT CTT GGC CTT TTG GCT TC	GGT GGT CTA GCA GGT TCT TGA AA
BNP	CAC CGC TGG GAG GTC ACT	GTG AGG CCT TGG TCC TTC AAG GTC ACT
Acta1	GAC TCC TCC CTC CCC TCT TA	GGT GTC TAG TTT CAG AGG CTG G
LC3b	CGT CCT GGA CAA GAC CAA GT	ATT GCT GTC CCG AAT GTC TC
Binp3	TTC CAC TAG CAC CTT CTG ATG A	GAA CAC CGC ATT TAC AGA ACA A
Gabarapl1	CAT CGT GGA GAA GGC TCC TA	ATA CAG CTG GCC CAT GGT AG
Sqstm1	CCA GAA TCG GAA GGG CCA A	TCA GCC TCT GGT GGG AGA TGT
Adamts15	TCA ACA TCG TGG TGG TCA AGG	CCG CAT AGG TCC TGT CTG GTG
Atxn3	AAG TCG CCA GGA AAT CGA CA	GCT GCT GCT GTT GCT TTT CAA
Btg2	GCA CTG ACC GAT CAT TAC AAA CA	CAC CTT GCT GAT GAT GGG GT
Daam1	CTC CGG GGC CAG TAG AGT AT	CCG GCT CCA TTG TCT GAA GT
Esrrg	CGG GCT CTG TCA AGG AAA CT	AGA GAA GCT CTT CTT CGT AGT GC
Jhdm1d	ACT GGC ACA GGC ATG ACT AC	CAT CGG CAC TTG GGA AGA CT
Lifr	CGG CCA AGA AAT CCA TAA CT	AAC GAA GTC GGA TCA TGA GG
Mfn1	ATT GCC ACA AGC TGT GTT CG	CTA GGG ACC TGA AAG ATG GGC
Pafah1b1	GCG AAC TCT CAA GGG CCA TA	CAT TGT GAT CGT GAC CGT GC
Slc38a9	GAT TGG CCT GCT GAG GAC TG	GCC CAG GGC TTA CTT CAT GT
Gapdh	ACCCAG AAGACTGTGGATGG	CACATTGGGGGTAGGAACAC
2610203C20	CTCCTGGAAGCTCTGCAACTATGC	TGGAGAGA AATGCTCTTTCTAACT
β-actin	CACAGTGTTGTCTGGTGGTA	GACTCATCGTACTCCTGCTT
